# The ongoing nutrition transition thwarts long-term targets for food security, public health and environmental protection

**DOI:** 10.1038/s41598-020-75213-3

**Published:** 2020-11-18

**Authors:** Benjamin Leon Bodirsky, Jan Philipp Dietrich, Eleonora Martinelli, Antonia Stenstad, Prajal Pradhan, Sabine Gabrysch, Abhijeet Mishra, Isabelle Weindl, Chantal Le Mouël, Susanne Rolinski, Lavinia Baumstark, Xiaoxi Wang, Jillian L. Waid, Hermann Lotze-Campen, Alexander Popp

**Affiliations:** 1 Potsdam Institute for Climate Impact Research (PIK), Member of the Leibniz Association, P.O. Box 60 12 03, 14412 Potsdam, Germany; 2 Institute of Global Health, University of Heidelberg, Heidelberg, Germany; 3 Institute of Public Health, Charité - Universitätsmedizin Berlin, Berlin, Germany; 4 INRAE, Agrocampus-Ouest, SMART-LERECO, Rennes, France; 5 Department of Agricultural Economics and Management and China Academy for Rural Development, Zhejiang University, Hangzhou, People’s Republic of China; 6 Department of Agricultural Economics, Humboldt-Universität zu Berlin, Berlin, 10099 Germany

**Keywords:** Environmental social sciences, Risk factors

## Abstract

The nutrition transition transforms food systems globally and shapes public health and environmental change. Here we provide a global forward-looking assessment of a continued nutrition transition and its interlinked symptoms in respect to food consumption. These symptoms range from underweight and unbalanced diets to obesity, food waste and environmental pressure. We find that by 2050, 45% (39–52%) of the world population will be overweight and 16% (13–20%) obese, compared to 29% and 9% in 2010 respectively. The prevalence of underweight approximately halves but absolute numbers stagnate at 0.4–0.7 billion. Aligned, dietary composition shifts towards animal-source foods and empty calories, while the consumption of vegetables, fruits and nuts increases insufficiently. Population growth, ageing, increasing body mass and more wasteful consumption patterns are jointly pushing global food demand from 30 to 45 (43–47) Exajoules. Our comprehensive open dataset and model provides the interfaces necessary for integrated studies of global health, food systems, and environmental change. Achieving zero hunger, healthy diets, and a food demand compatible with environmental boundaries necessitates a coordinated redirection of the nutrition transition. Reducing household waste, animal-source foods, and overweight could synergistically address multiple symptoms at once, while eliminating underweight would not substantially increase food demand.

## Introduction

Dietary patterns are shifting world-wide, yet not synchronous, from scarce, plant-based diets with fresh and unprocessed foods towards affluent diets high in sugar, fat, and animal-source foods, featuring highly-processed food products^1^. While the prevalence of undernourishment is persistent in absolute numbers due to population growth^2^, this “nutrition transition”^1, 3^ causes a relative shift in public health challenges from undernutrition-related infectious diseases and neonatal disorders towards overconsumption related chronic diseases such as diabetes and cardiovascular diseases^3, 4^. Currently, suboptimal diets are the leading global health risk with an estimated yearly loss of 255 million attributable disability-adjusted life years (DALYs)^5^. Adopting healthier diets could annually avoid 11–12 million premature deaths among adults^6^.

Global food demand is shaped by this nutrition transition, but also by population growth, changing demographic structure, lower levels of physical activity, and increasing household food waste. This growing demand for food is the leading driver of agricultural production and thereby also the main interface between human society and the environment^12^. Agriculture covers one third of global land area^7^ and is responsible for 70% of anthropogenic blue water use^8^. The food system is responsible for 21–37% of anthropogenic greenhouse gas emissions^9^. Agriculture also increased the polluting release of nutrients into the environment, being the dominant driver behind the quintupling of nitrogen surplus on land systems relative to preindustrial times^10^. Finally, agriculture strongly contributes to air and water pollution, soil degradation, antibiotic resistances, new pathogens, as well as biodiversity loss.

Undernutrition, overnutrition and food-related environmental pollution co-exist in all world regions, are affected by common drivers and require common solutions, but have too long been analyzed in academic silos^11, 12^. Consumer behavior, including dietary choice and food wasting behavior, is central to all three problems, and any policy designed to bring about behavioral change in these areas has to be carefully evaluated in regard to trade-offs and synergies. The Lancet-Commission on the “Global Syndemic of Obesity, Undernourishment and Climate Change” stresses that this synergy of three epidemics represents the uppermost health challenge of the twenty-first century^11^, and urges the scientific community to develop modelling studies to provide an evidence-base on the Global Syndemic for policy makers and to create collaboration across the different communities.

Our study therefore compiles a comprehensive international database of food consumption, and estimates different symptoms of the Global Syndemic within a consistent framework, allowing for integrated analysis of global health, food systems and environmental change. Our central research question is: How did various food-consumption related symptoms of the Global Syndemic develop worldwide over the last decades, and what are the outcomes if the observed nutrition transition continues into the future? The analyzed symptoms include the prevalence of underweight, overweight and obesity, body height, caloric intake, food waste in households, dietary composition and total demand for food and animal-source foods.

Our estimates are based on an open-source model (see “Materials and methods”), which is used to integrate available data for the period 1965–2010, to complement data gaps in historical data, and to project future scenarios for the period 2010–2100 based on the trajectories of population growth, demographic change and income development of the five Shared Socio-Economic Pathways (SSPs)^13^. Our assessment starts by projecting the prevalence of underweight, overweight and obesity. For the first time, we provide future projections of inter-country and intra-country distributions of body mass index by age-class and sex. Next, as a proxy for stunting, we provide the first international projection of body height. Combining the estimates of body mass index and body height with physical activity data and with projections of changing demographic structure allows us to estimate food energy requirements, which are a good proxy for food intake assuming a stable-state of body mass. In contrast to food demand estimates^7^, being the sum of food intake and food waste, isolated food intake estimates are not available in public statistics but can only be estimated indirectly. The conventional approach of applying uniform regional food waste shares^14^ to isolate food waste from food intake are satisfactory for estimating environmental mitigation potentials^15^, but are of insufficient quality to derive food intake estimates for epidemiological analysis, where small energy misbalances have large public health implications, and where under- and overintake are equaling out. Our bottom-up estimate of food intake is here more nuanced and also provides estimates for subpopulations within countries. The combination of food intake estimates with food availability data allows for a top-down estimation of food waste in households. Similar previous estimates of food intake did either not account for future trajectories^16^ or used static BMI estimates^17^.

Moreover, our anthropometric approach allows to empirically differentiate how much of the rising food demand can be attributed to population growth, ageing, increasing height and body mass index, declining physical activity and increasing food waste. The potential of mitigation measures which address consumer’s behavior, such as food waste reduction^10, 18^ or obesity prevention, can therefore be estimated with more nuance. Finally, our study also analyses and projects the dietary composition using a nested demand model that guarantees consistency of individual food groups with total calories. Compared to previous model versions with less food groups^19, 20^, our model now considers four major food groups of epidemiologic and environmental relevance: animal-source foods; empty calories (the calories from oils, sugar and alcoholic beverages^21^); vegetables, fruits and nuts; and staples. Combining our per-capita estimates with population projections also allows for projections of total food demand, total food waste, as well as the demand for resource-intensive and environmentally polluting animal source foods. Using a novel methodology for food demand projections that includes anthropometric dynamics, as well as estimating all our elasticities independently provides a valuable validation for the existing food demand projections which often use the same parameters for their elasticities^22^.

## Results

### Undernutrition declines in relative terms but stagnates in absolute numbers

**Figure 1:**
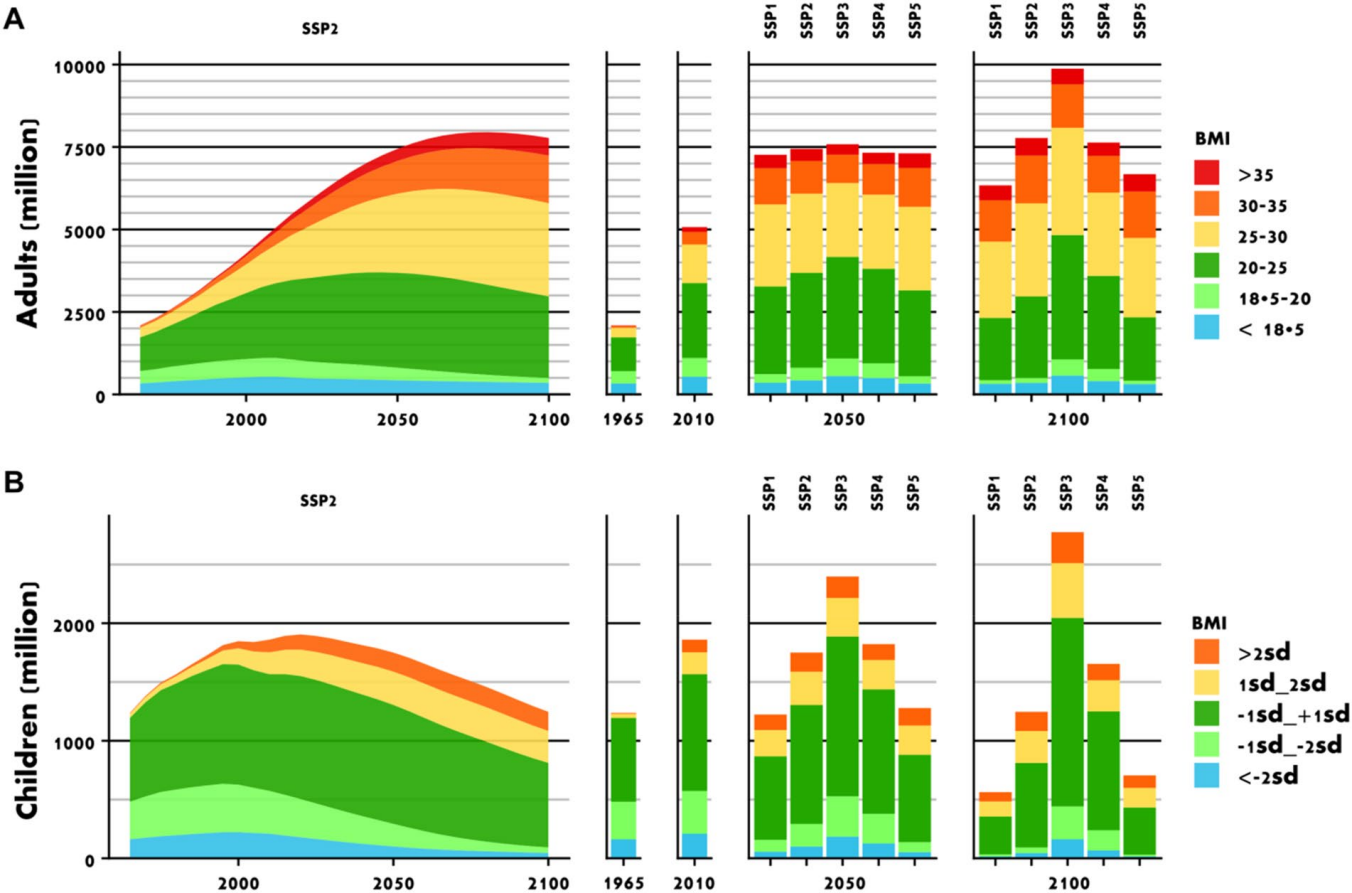
The prevalence of underweight decreases in the world population, while overweight and obesity increase. The figure shows the world population by body mass index (BMI) for adults (15+ years) (**A**) and children (0–14 years) (**B**) over the period 1965–2100 for different population scenarios of the Shared Socioeconomic Pathways (SSPs) in million people. The left side is the middle of the road scenario SSP2, the right side provides comparison to the other SSPs for 2050 and 2100. Colors categorize by absolute body mass index (BMI) for adults, and by standard deviations from WHO growth standards for children. Blue colors indicate underweight, yellow overweight, and red obesity.

Our projections highlight that current efforts in combating undernutrition will fail to achieve the Sustainable Development Goal to end hunger (SDG2) by 2030 as underweight remains a persistent problem affecting several hundred million people (Fig. 1). The prevalence of underweight in a middle-of-the-road scenario (SSP2) only declines from 744 million in 2010 (11%) to 528 million (6%) and to 394 million (4%) by 2100. Estimates for the entire range of SSP scenarios with different trajectories of demography and per-capita income vary from 383 to 741 million (4–7%) in 2050 and from 330 to 733 million (4–6%) in 2100. In 2010, male children are disproportionally affected by underweight (13%), while in our projections for 2050 next to male children also older people (60+) of both sexes have a slightly higher prevalence (7%) than the average.

**Figure 2:**
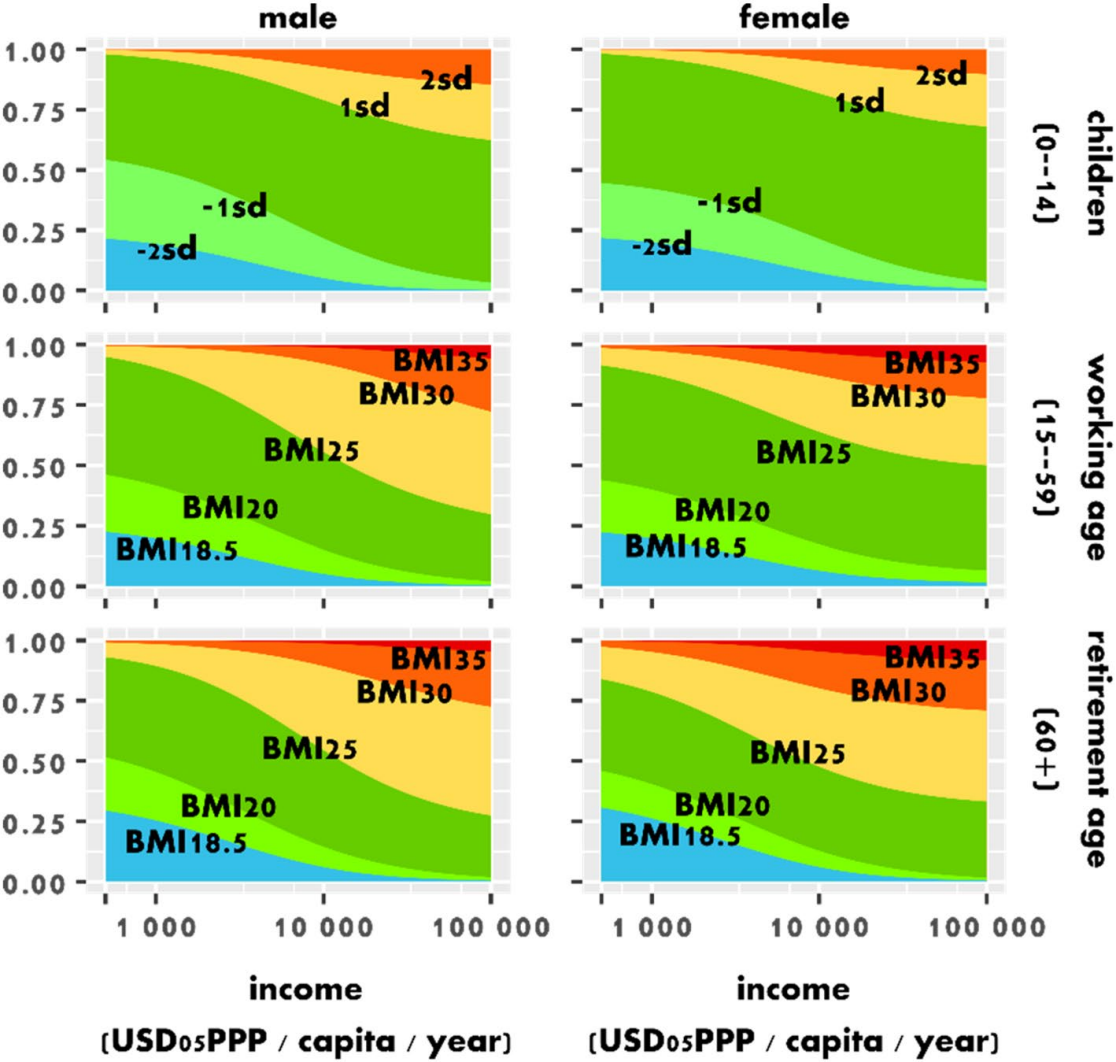
With rising per-capita income, underweight declines and overweight increases, while the share of people with a healthy Body Mass Index (BMI) declines. The figure shows the estimated isoquants for the proportions of the population with specific Body Mass Index (BMI) by per-capita income (in US Dollar 2005 in purchase power parity) for males (left) and females (right) divided by age-groups into children of 0–14 years (top), working age adults of 15–59 years (middle) and retirement age adults of 60+ years (bottom). For children, we use standard deviations from WHO growth standards. Blue colors indicate underweight, yellow overweight, and red obesity*.*

**Figure 3:**
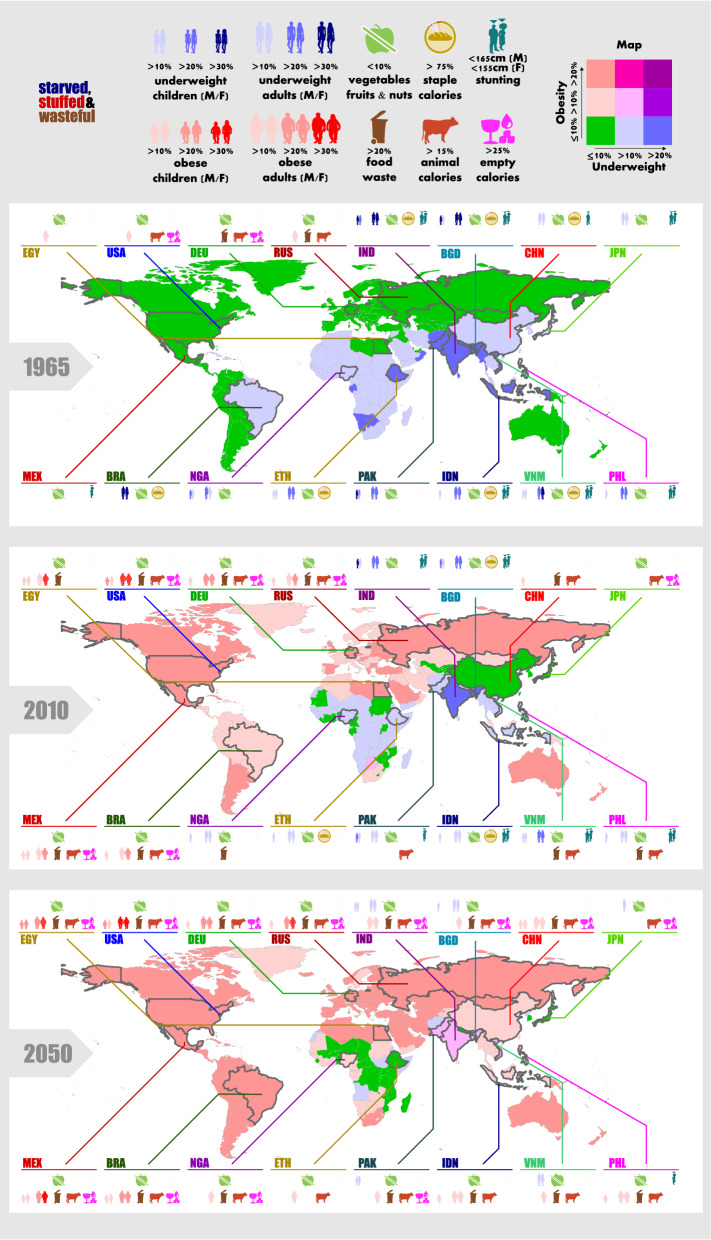
Unbalanced diets: The shift from scarcity to overconsumption. The map colors show the prevalence of underweight and obesity in the population. For the 16 most populous countries, symbols indicate further details on anthropometrics, dietary composition and food waste. Country abbreviations are ISO3-country-codes. Estimates for 2050 are model projections. For 1965 and 2010, reported data is complemented with model estimates for missing data. Body mass index estimates for 1965 were complemented as reported data only starts in 1975. Dietary composition data had to be complemented for some major countries without reported data such as the Philippines or the Democratic Republic of the Congo. Food waste estimates are all model projections.

**Figure 4:**
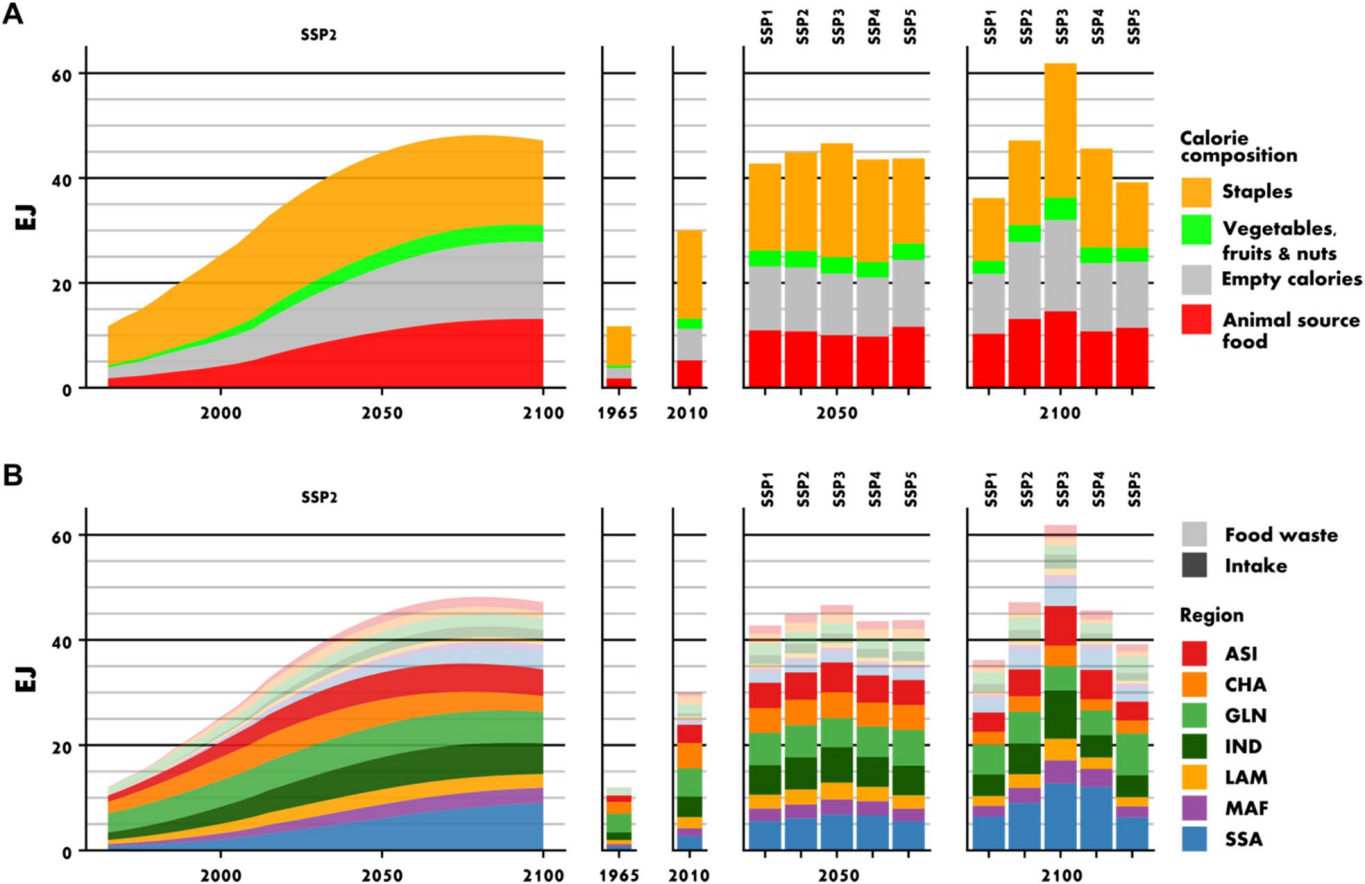
Global food demand is expected to strongly increase. Demand for animal-source foods and demand for empty calories increase over-proportionally (**A**). The largest increase of global food demand is projected for Africa (**B**). The figure shows total food demand by main food groups (**A**), and by world regions split by intake and food waste (**B**). *ASI* Rest of Asia, *CHA* China, *GLN* Global North, *IND* India, *LAM* Latin America, *MAF* Middle East and Northern Africa, *SSA* Sub-Saharan Africa. See Supplementary Information 1 for the country-region mapping.

When economic growth sets off in low income countries, the nutrition transition commences and underweight declines (Fig. 2). Both increasing body mass index (BMI) and demographic change towards more adults lead to increasing per-capita food intake, which more than countervails any intake reduction due to declining physical activity levels (PAL). The relative decline in undernutrition is accompanied by higher dietary diversity, with rising consumption of animal-source foods, empty calories, as well as vegetables, fruits and nuts. In contrast, the consumption of staple foods stabilizes (Figs. 3, 4B). Higher food intake and more diverse diets also lead to continuously rising body height. From 1965 to 2010, average global adult height increased from 167 to 169 cm for males and from 155 to 157 cm for females. In 2050, height may reach 171 cm for men and 158 cm for women. Even in 2050, average body height still varies between countries from 151 to 170 cm for females and from 162 to 182 cm for men.

### The obesity epidemic advances

Already while undernutrition just starts to decline in middle income countries, overweight and obesity begin to spread (Fig. 2). In our middle-of the road scenario, overweight and obesity increase from 1993 million (29%) in 2010 to 4135 million (45%) in 2050 and 5018 million (56%) in 2100. Varying the scenarios of demography and income between SSP1—5 provides a range from 3842 to 4430 million in 2050 and from 4331 to 5637 million in 2100. Similarly, the number of obese increases from 636 million (9%) in 2010 to 1493 million (16%) in 2050, and 2052 million (23%) in 2100. While only 1% of children were obese in 1965, obesity reached 6% in 2010 and may further increase to 9% in 2050 and 13% in 2100, again violating the targets of SDG2 to end all forms of malnutrition. Among children, overweight and obesity is higher for males than for females. Among adults, overweight is more prevalent among working-age men, but obesity is similar for males and females, and higher for women aged 60+ (Figs. 2, 3). In the absence of behavioral change, our results show a future that is characterized by overweight and obesity of pandemic magnitude. This future pathway stands in opposition to the SDG2 target to end all forms of malnutrition and places an enormous burden on public health. In the U.S. alone, costs of diagnosed diabetes are estimated at 327 billion US$_2017_ in the year 2017^23^.

### The unceasing growth of food demand

We estimate that global food demand, which increased from 12 Exajoules (10^18^ J, EJ) in 1965 to 30 EJ in 2010, will further increase and may reach 45 (43–47) EJ in 2050 and 48 (36–62) EJ in 2100 (Fig. 4). In particular SSP3, assuming high population growth, leads to a very high demand. Within the period 1965–2010, the largest increase in global food demand came from Asia and Northern Africa, while in the future in particular India and Africa will drive the increase.

The prevalence of underweight reduces the global food energy demand by only about 1% in 2010 and 2050 (see Table 1), as already a small reduction in energy intake is sufficient to shift the metabolic equilibrium to underweight, and as only a share of the world’s population is affected by underweight. Increasing the physical activity levels of the part of the population with sedentary lifestyles to moderate activity, in line with WHO recommendations, would increase food demand by 5–6% in 2010 and 2050. In contrast, when all overweight and obese people would be normal-weight, intake would be reduced by 4% in 2010 and 7% in 2050. Yet, a reduction of food waste offers the highest reduction potential. Food demand exceeds intake by 25% in 2010, and by 33% in 2050 in the SSP2 baseline.

**Table 1:** Hypothetical change in global food demand if (from left to right) all underweight people were normal-weight, all overweight people were normal-weight, all physical inactive people had moderate physical activity levels, or food was not wasted in households.

				
2010	+ 1.4%	− 4.4%	+ 5.2%	− 24.9%
2050	+ 0.7%	− 6.8%	+ 5.5%	− 33.2%

Numbers for 2050 are from the SSP2 scenario.

**Figure 5:**
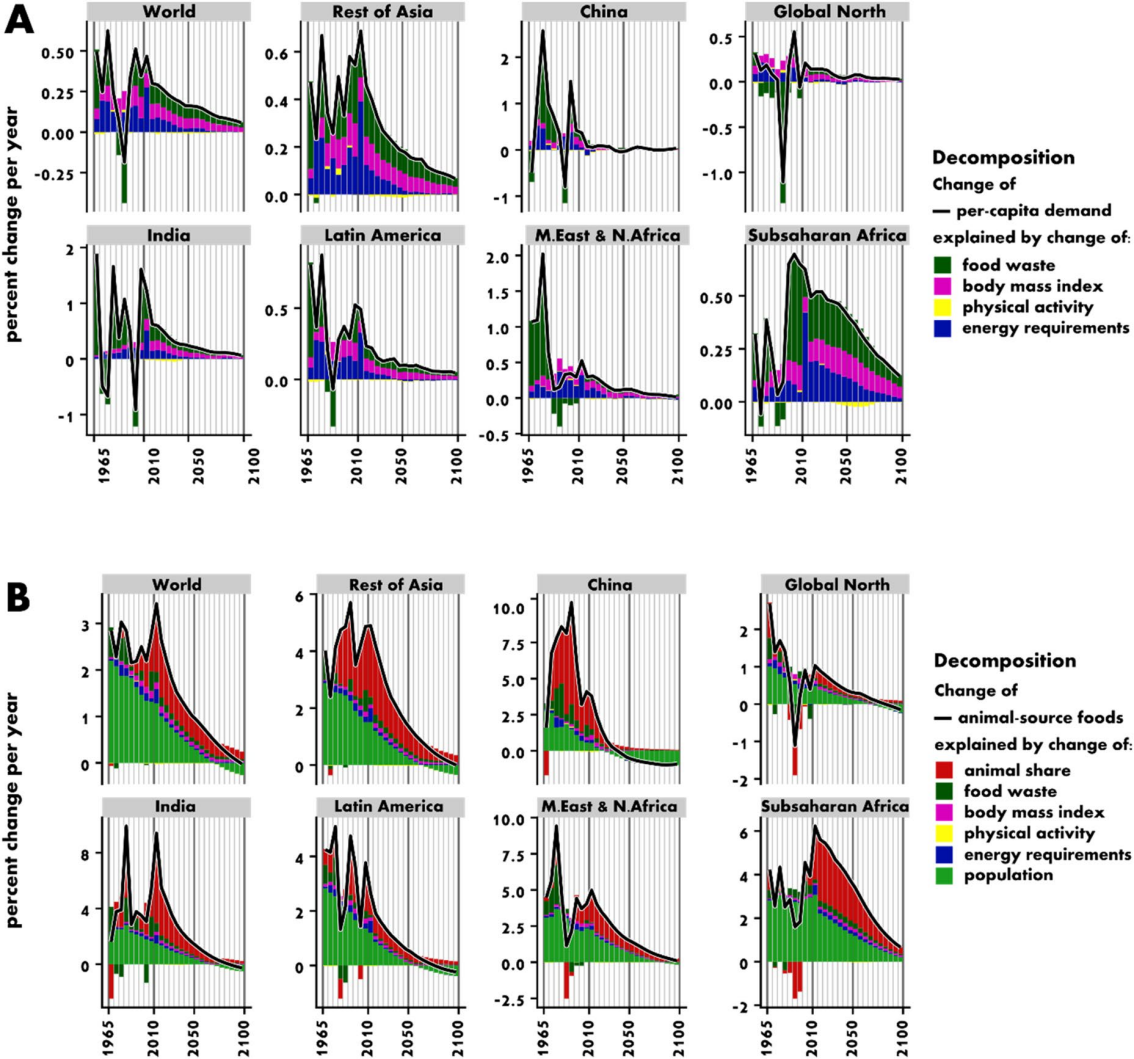
Growth in per-capita food demand is driven to similar extents by increasing body mass index (and connected under- or overconsumption), higher food waste and rising food energy requirements (dependent on age, sex and height for a normal-weight BMI) (**A**). Total demand for animal-source foods is mainly driven by population growth and a higher share of animal-source-foods in diets (**B**). The figure shows a decomposition of growth rates based on the decomposition method described in Supplementary Information 2, section S11 for different world regions (see Supplementary Information 1 for the country-region mapping). Black line indicates net growth.

Decomposing the per-capita food demand into its underlying processes (Fig. 5A), we can see that rising food energy requirements (i.e. the energy requirements for a normal-weight population with changing demographic structure and body height) have a similar influence as the increase of food waste and of overconsumption connected to higher BMI. The decline in PAL has only negligible effects.

We estimate that the demand for resource-intensive animal-source foods increases at even higher pace than total food demand, with the share of animal-source calories in total calories rising globally from 18% in 2010 to 24% (22–27%) by 2050 (Fig. 4A). Our decomposition analysis (Fig. 5B) reveals that the expected doubling of animal-source foods can be attributed mostly to population growth and an increasing share of animal calories in diets, while rising food waste, overconsumption connected to increasing BMI, reduced PAL or rising food energy requirements have less influence on future changes in the demand for animal-source foods.

Global results with country-level resolution for underweight, overweight, obesity, intake, food demand, food composition, as well as male and female body height for the period 1965–2100 can be found in the Supplementary Information 1. To create a comprehensive dataset for follow-up impact studies, we filled data gaps in the model driver input data (Supplementary Information 2, section S8). Subsequently, we applied the model not only for future projections, but also filled data gaps in historical data, which was incomplete in terms of temporal and spatial coverage. Data gaps included for example all BMI data for 1965 and 1970, as well as dietary composition data for some major countries without reported data such as the Philippines or the Democratic Republic of the Congo (see Supplementary Information 2, Table S1).

### Comparison to out-of-sample data and to other studies

A five-fold cross-validation of our model can be found in Supplementary Information 2, section S9. It shows that our model is robust to outliers, and uncalibrated estimates have a coefficient of determination (R^2^) with out-of-sample selections of reported data of 0.418–0.722 for the year 2010, depending on the evaluated indicator. When calibrating our model to 1975, the first year with a complete set of reported data also for BMI distribution data, the match of our results with out-of-sample reported data of 2010 was further improved to an R^2^ of 0.542–0.897. In contrast to our previous model version^19^ which relied on a statistical model with time-dependent parameters, the model of this study shows a better fit with reported data for total and animal-source calories without the need of time-dependent model parameters (see Supplementary Information 2, section S10).

In our middle-of-the-road scenario, global food demand increases by + 65% until 2050 relative to 2005 which is within the range of multiple other projections, such as the rise by + 54% estimated by the FAO^24^, or the estimated + 70% by Bijl et al.^25^. For global livestock calorie demand we estimate an increase by + 108% relative to 2005, which is again higher than FAO’s projections of + 76%^24^ and close to the estimate of + 110% by Bijl et al.^25^. An extensive comparison of our projections to other studies^19, 24–26^, including regional and commodity-specific estimates, is presented in Supplementary Information 2, section S10.

## Discussion

Our modeling study shows that the nutrition transition is an ongoing and unchecked global process. Popkin’s theory^1^ of a transition of dietary patterns from “famine” to “receding famine” to “degenerative diseases” can be supported, yet his hypothesis of a development towards a pattern of “behavioral change” with less processed food and a decline in obesity is yet, 25 years later, not observable^27^. In contrast, our analysis shows that obesity still rises even in high-income countries, while the share of people with a healthy BMI is declining. In low-income countries, normal weight individuals still make up three quarters of the population, but with rising income the share of normal weight population continuously declines. In middle-income countries, patterns of underweight and overweight often coexist. We show that in particular during the transition from a lower-middle to an upper-middle income country, dietary composition shifts towards very high consumption of animal-source foods and empty calories, while the consumption of vegetables, fruits and nuts stagnates at low levels. Energy-dense animal-source foods and empty calories become unhealthy when consumed in high quantities and replace healthier food groups. Within our empty calorie group, mono- and polyunsaturated fats have little dietary risk and are often beneficial, while saturated fats and trans fats are already consumed excessively^6, 28^. Our finding of increasingly unbalanced diets is consistent with Imamura et al.^29^ who found that the increased consumption of healthy food items has been outpaced by the increase of unhealthy items. Indeed, balanced diets are more prevalent in lower-middle income countries than in high-income countries.

Without a paradigm change in food policy, the full consequences of the Global Syndemic will unfold in the coming decades. Our projections for underweight show that the SDG target of zero hunger remains continuously out of reach, while the obesity epidemic and malnutrition further aggravate. Multiple studies (Supplementary Information 2, Table S5) have investigated the environmental consequences of future scenarios with comparable food demand growth as our study. It is evident that further demand growth, as projected by our study, will result in unsustainable land expansion, water withdrawals, nutrient pollution, greenhouse gas emissions and biodiversity loss. Several studies showed that without a substantial shift from animal- to plant-based products and a strong reduction of food waste, the agricultural system cannot return into the sustainable “planetary boundaries” that define a safe operating space for humanity^10, 18, 30^.

Our study shows how the rising food demand is the consequence of changes in human population, demographic structure, physical activity levels, body height, body mass index and food waste. Separating these effects accurately is not only important for estimating the potential impacts of reducing food waste or obesity on food demand and environmental pollution (Table 1). It is also crucial for agro-economic modelling studies in which the number of undernourished is back-calculated from per-capita food demand^31^, as the latter can easily lead to false attribution. For example, the difference in healthy food energy requirements (BMI 20–25) between Sub-Saharan Africa (2025 kcal per capita per day in 2010) and the Global North (2304 kcal/capita/day) or China (2323 kcal/capita/day) are in the order of 250–300 kcal per capita per day due to a higher share of children and lower height in Sub-Saharan Africa. In comparison, the difference in daily energy intake between an underweight (BMI < 18.5, 1893 kcal/capita/day) and the food energy requirements normal-weight population in Sub-Saharan Africa is just 100–150 kcal. None of the available global demand projections takes the effects of changing demographic structure, body height, BMI, or physical activity explicitly and simultaneously into account for their calorie projections. Any back-calculation of these estimates to people at the risk of hunger or obesity is therefore prone to misattribution or inconsistencies.

Our modelling exercise is also subject to a number of limitations.

First, our anthropometric food intake estimates exceed the FAOSTAT dietary energy availability in some low-income countries. Different metabolic equations, taking into account also body height^32^ or outdoor temperature^33^, did not alter this finding. Various possible explanations for this mismatch are discussed in the Supplementary Information 2, section S6.

Second, our estimates are uncertain for countries that reach very high income levels (> 50,000 USD per capita). In 2050, roughly one billion people live in such countries in SSP2, but little reported data exists for dietary patterns at comparable incomes. Alternative interpretation of historic data can result for example in a falling share of animal-source foods for very high income levels^10, 34^. Our model is therefore best suited for storylines that assume a continuation of materialistic lifestyles such as SSP2, SSP3, SSP4 and SSP5, while it is less suited to simulate a scenario like SSP1 that assumes a preference change towards more sustainable lifestyles^13^. Similarly, our choice of the functional form for the regressions assumes a saturation of the prevalence of overweight and obesity for very high incomes at a level close to current maximum prevalence. Yet, overweight and obesity are still increasing in high-income countries and the actual level of saturation is not foreseeable. Our estimates of overweight and obesity in high-income countries are therefore conservatively low.

Third, while our model newly includes anthropometric and demographic dynamics, the only considered socio-economic driver is the country-average per-capita income. This is a simplification as many other factors affect diets and food demand, such as intra-country inequality, education, urbanization, globalization, (super)market access, advertisement, food prices, policy measures, and the strength of the food industry^1, 3, 25, 35–38^. However, most of them are very collinear with per-capita income. The model driver should therefore not be misinterpreted as household income, but as a proxy for aggregate socio-economic development^3^. Nevertheless, economic development as aggregate driver can only insufficiently explain certain dynamics. For example, the share of obese adults shows a positive time-trend beyond the income trend (Supplementary Information 2, Figs. S4–S6). This suggests explanatory dynamics that are independent of a country’s current economic development, such as global technological development, social globalization, or the international influence of Western food industry. Without representing these continuous dynamics, our model likely underestimates future obesity.

Future model development should try to include further plausible explanatory variables for which reliable future projections exist, such as urbanization or climatic variables^38^ to reduce the residual error. Including food prices becomes relevant when ambitious policy scenarios shall be investigated^39^, but requires more robust food price projections^40^. Also the inclusion of time lags could be explored to capture sticky preferences, slow changes in the built environment and epigenetic dynamics^27^. Finally, the parametrization of the model should be updated when new reported data becomes available to reduce in particular the uncertainty for countries with very high incomes where few observations exist so far.

Uncertainties of projections differ by the process driving the increasing demand. Two major dynamics, the connection to population growth and to rising food requirements of an ageing and taller population (Fig. 4), are well-established based on biophysical relationships, and uncertainties mainly depend on the range within underlying demographic projections. By comparison, the correlation of economic development to rising body-mass index, increased food waste or changing dietary composition depend on the interaction of complex socio-economic processes. While this analysis finds that the correlations to per-capita income have been relatively stable over time and similar across countries, these social dynamics could also be subject to disruptive change, e.g. due to technological or social innovation or due to policy intervention.

## Policy implications

So far, forward-looking national assessments of diets and anthropometrics are absent for most countries^41^, leading to status-quo bias in policy strategies, infrastructure expansion and economic investments. Here, our comprehensive projections allow policy-makers to build expectations on the high speed and large magnitude in which diverse symptoms of the nutrition transition may spread, in particular in current low- and medium-income countries. Going beyond our global database, national assessments are urgently needed, and could include more detailed statistics and scenarios, e.g. differentiating social milieus, or exploring policy interventions that are tailored to the local context. For long-term projections under continuous economic growth, national studies however face the problem to extrapolate out of the domain for which observed national data exists. Here, our cross-country analysis could provide orientation to inform national assessments about the development pathway of other countries.

Our empirical analysis substantiates that current food policy has not managed to achieve a paradigm change of the nutrition transition. So far, no country can serve as an example for a successful policy-induced reduction of obesity^27^, animal-source foods or food waste. There is no evidence that individual decisions or private sector action will suffice. Instead policy leadership is required, implementing a combination of multiple integrated policy instruments^27, 35^.

Discussed policy measures include pollution taxes to price environmental externalities, consumption taxes to internalize public health and community care costs, marketing restrictions for unhealthy and polluting food items, nutrition education such as cooking classes or school gardens, public provision of healthy and sustainable food in canteens, raising public awareness, obligatory food labelling, and regulatory policies such as the ban of trans-fatty acids. Politics can also shape the wider food environment that leads to behavioral change^27, 36^, e.g. through the support of care work by part-time schemes or through providing family planning services.

Recognizing the syndemic nature of obesity, undernutrition and environmental pollution can help to efficiently coordinate the transformation of the food system and to mobilize the necessary momentum of change. By quantifying the potential trade-offs and synergies (Table 1), we show that eliminating underweight would not lead to a substantial increase in food demand. Increasing physical activity to moderate levels has a higher impact, but could be more than compensated for by obviating overweight and obesity. The highest synergies for reducing multiple symptoms of the nutrition transition could be obtained by substituting animal-source foods and by reducing household waste. A reduction in animal-source foods reduces obesity and environmental pressure. Moreover, reducing food waste and animal products would lower food prices and help to fight undernutrition^42^. Given our projected future nutrition trajectory of current low-income countries, international aid should shift its priorities anticipatorily from investing in supply chains of animal products and processed products towards investments in horticultural supply chains^43^. Research and policy action therefore need to be integrated between disciplines and policy domains, starting with the formulation of integrated national nutrition guidelines, an outlook of long-term challenges to the food system, and an agenda to achieve behavioral change.

In conclusion, our study shows that current trends of the nutrition transition are not in line with achieving the SDGs in respect to multiple targets for food security, public health and environmental sustainability and will lead to the transgression of multiple planetary boundaries. Research and policy makers have to become proactive to restructure the food system towards prevention and mitigation of these impacts, and build the capacity within the health system to handle the shifting risk factors in MICs. Policy action focused on consumption behavior should holistically target all forms of malnutrition as well as environmental impacts, as there are synergies and trade-offs, most importantly in respect to animal-source foods and food waste. Forward-looking national dietary assessments on this syndemic can benefit from the orientation that our cross-country analysis provides. Our comprehensive open dataset and model can support this planning process and provides the interfaces necessary for integrated studies of public health^4, 6, 31, 44^, food systems^22, 25, 45^, and environmental change^10, 18, 46, 47^ on a global scale.

## Materials and methods

### Food demand model

**Figure 6:**
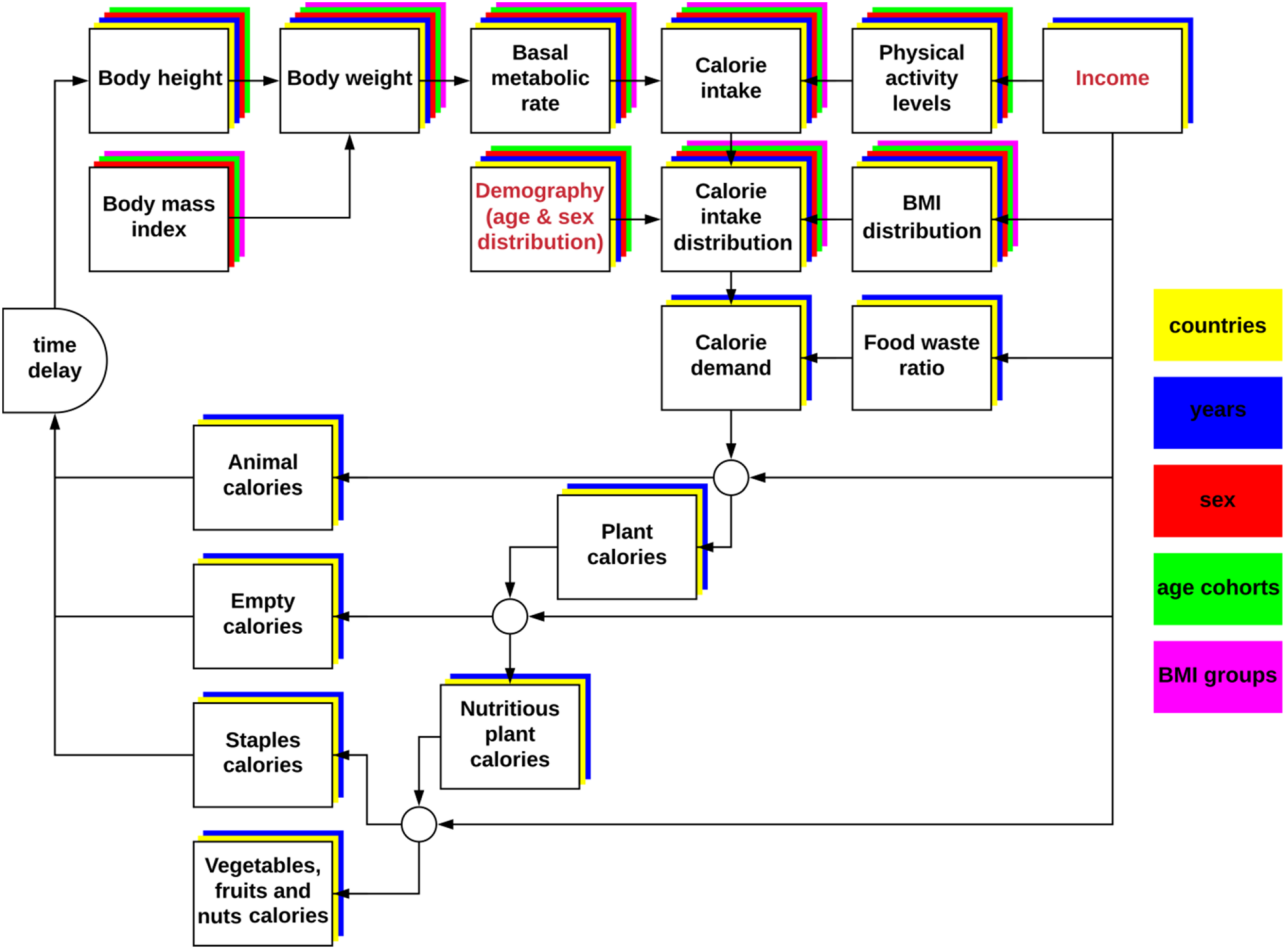
Model design. Red font indicates model drivers. Colored layers indicate the dimensionality of the variables.

Our open-source food demand model^48^ is designed for long-term scenarios of food intake, dietary composition, body mass index (BMI) distribution, body height and food waste. The simulations are carried out in 5-year time steps “*t*” from 1965 until 2100 for the 249 ISO 3166-1 countries and territories “*c*”. Depending on data availability, some variables further distinguish sub-populations based on sex “*s”* (male/female), age-cohorts “*a*” (20 five-year cohorts from 0 to 100+), or BMI classes “*b*” (six BMI ranges for adults and five BMI ranges for children). Figure 6 provides an overview of the model design and the sequence of estimations carried out within a 5-year time step of the period 1965–2100.

We use regression analysis with historical data to estimate the parameters (indicated in the following as Greek letters) for each functional relation (See Supplementary Information 2, section S1 for regression method, and Supplementary Information 1 for statistical indicators). The model is applied for forward-looking scenarios using only income $Y_{c,t}$ and population $P_{a,s,c,t}$ projections as drivers.

Compared to conventional econometric approaches that estimate per-capita food demand as a direct function of per-capita income, we consider also explicitly the role of anthropometrics in determining food demand. We combine BMI and body height to determine weight for each age-sex combination. As more body cells require more energy for maintenance, the basal metabolic rate (BMR) is strongly correlated to body weight. Further, the BMR differs by age and sex, which is why we apply the metabolic Schofield equations for each age–sex combination separately to determine BMR as dependent variable based on body weight as independent variable. We multiply the BMR with the physical activity level multiplier (PAL) to determine the total energy burned. This energy has to be replenished through food intake to maintain body weight, and the feeling of hunger and saturation balance food intake within a narrow band. Certain modulations of food intake are possible by higher or lower BMI and PAL. In our model, BMI distribution and PAL are therefore functions of per-capita income. The reason to estimate income elasticity for BMI distributions rather than for intake distributions is that the quality of BMI data is higher than food intake estimates which can only be inferred indirectly with residual errors, while the BMI regressions can be parametrized with a lower information loss. As the model is deterministic, the order of estimation does however not imply a direction of causality.

We start with the estimation of body height of the male and female age cohort 15–19 years (see Supplementary Information 2, section S2 for a detailed description of height estimates). Next to genetics, height is strongly connected to dietary quality, in particular to dietary diversity, nutrient density, protein intake and the consumption of animal-source foods by the mothers and the children^49, 50^. To parametrize our height equation

1 $$H_{15 - 19,s,c,t} = \alpha_{s} G_{c,t^{\prime}}^{{\beta_{s} }} .$$

we regress past observations of sex-specific height of young adults^51^ $H_{15 - 19,s,c,t}$ with per-capita food demand for the sum of calories from animal-source foods, pulses and oils $G_{c,t^{\prime}}$ from FAOSTAT^7^. To account for the growing period and to avoid circularities in the model, we use the preceding three 5-year timesteps “t’”, covering the time-span of approximately one year before pregnancy until the completion of the 14th life year. Older adult age-cohorts keep the height that was estimated when they reach adult age. For children, we estimate height by scaling the WHO growth standards for children^52^ and adolescents^53^ with the same factor by which the 15–19 years age-cohort diverges from the WHO growth standards for 18-year old adults^53^.

Next, we distinguish different BMI groups within the population. Each BMI group is assigned an average BMI $B_{b,a,s}$. $B_{b,a,s}$ and $H_{a,s,c,t}$ are used to estimate body weight $W_{b,a,s,c,t}$:

2 $$W_{b,a,s,c,t} = B_{b,a,s} H_{a,s,c,t}^{2} .$$

We estimate PAL $A_{a,s,c,t}$ for male and female *children* (0–14 years), *working-age adults* (15–59 years) and *retirement-age adults* (60+ years) sub-populations. With economic development, PALs usually declines due to lower manual labor in agriculture and industry^37^. Also, in retired adults inactivity is much more common than in younger adult age-groups^54^. Due to limited available data, we construct a dataset of observed physical inactivity levels^55^, which is completed with a rule-based approach using per-capita income^56^, age and gender (Supplementary Information 2, section S3). Then, inactivity levels are translated into *PALs* by applying sedentary-lifestyles multipliers for inactive people, and active-lifestyle multipliers for the remainder^32^.

Applying the Schofield equations^32^ (Supplementary Information 2, section S4), we derive the basal metabolic rate (*BMR*) of male and female age-cohorts, which expresses the food energy intake of a resting human, and which is dependent on body weight, sex and age. For comparison and discussion, we also apply the Schofield equations that use body weight, height, sex and age^32^, as well as a set of equations by Froehle et al.^33^ that estimates BMR based on weight, sex, age, and temperature.

The BMR is multiplied with PAL to estimate the *food intake*:

3 $$I_{b,a,s,c,t} = (\gamma_{a,s} W_{b,a,s,c,t} + \delta_{a,s} )A_{a,s,c,t} .$$

Next, we estimate which shares of the population have a certain BMI (Supplementary Information 2, sectionS5), again distinguishing between male and female *children* (0–14), *working* (15–59) and *retired* (60+) populations. We regress the BMI-group, age-group and sex-specific population shares $S_{b,a,s,c,t}$^57^ with per-capita income $Y_{c,t}$^56^ to parametrize the functions $f_{a,s} ()$ (Fig. 2):

4 $$S_{b,a,s,c,t} = f_{a,b,s} \left( {Y_{c,t} } \right).$$

Food intake is aggregated from sex and age-group specific data to country totals using demographic data^58^, and adding an additional food energy requirement *N* for pregnant and lactating women^32^, which is estimated based on the number of newborns $P_{0 - 4,s,c,t}$ in a 5-year timestep:

5 $$I_{c,t} = \frac{{\mathop \sum \nolimits_{b,a,s} I_{b,a,s,c,t} S_{b,a,s,c,t} P_{a,s,c,t} }}{{\mathop \sum \nolimits_{a,s} P_{a,s,c,t} }} + \mathop \sum \limits_{s} NP_{0 - 4,s,c,t} /5.$$

*Food demand* on country level (defined as the calorie availability estimated by FAOSTAT) should be larger than *food intake* because food waste at household level is included in FAOSTAT estimates^17, 37^. To estimate food waste $X_{c,t}$, we compute the ratio of *food demand* $D_{c,t}$^7^ and *food intake* $I_{c,t}$ using a regression with *per-capita income* $Y_{c,t}$ (Supplementary Information 2, section S6).

6 $$\frac{{D_{c,t} }}{{I_{c,t} }} = \frac{{\varepsilon Y_{c,t} }}{{\zeta + Y_{c,t} }} + 1,$$

7 $$X_{c,t} = D_{c,t} - I_{c,t} .$$

Dietary composition considers four food groups which we selected based on criteria of relevance for population health and environmental pressure as well as intra-group substitutability: *animal calories* (the calories from animal-source foods including seafood), *empty calories* (the calories from oils, sugar and alcoholic beverages^21^), *vegetables, fruits and nuts* calories, and *staples* (the calories from remaining foods, mostly cereals, tubers, roots and pulses). To estimate the dietary composition while remaining consistent with total calorie consumption, we decided for a nested tree structure (Supplementary Information 2, section S7), where each food group is divided into two calorie demand shares^7^ as a function of per-capita income^56^ $Y_{c,t}$. First we divide total food demand into *animal calories* $L_{c,t}$ and *plant calories* $\left( {D_{c,t} - L_{c,t} } \right)$

8 $$L_{c,t} = \frac{{\eta Y_{c,t} }}{{\theta + Y_{c,t} }}\,D_{c,t} .$$

We further subdivide the plant calories into *empty calories* $E_{c,t}$ and *nutritious plant calories* $\left( {D_{c,t} - L_{c,t} - E_{c,t} } \right)$:

9 $$E_{c,t} = \frac{{\iota Y_{c,t} }}{{\kappa + Y_{c,t} }}\left( {D_{c,t} - L_{c,t} } \right),$$

and finally split into *fruits, vegetables and nuts* $V_{c,t}$ and the remaining *staples *($R_{c,t}$):

10 $$V_{c,t} = \frac{{\lambda Y_{c,t} }}{{\mu + Y_{c,t} }}\left( {D_{c,t} - L_{c,t} - E_{c,t} } \right),$$

11 $$R_{c,t} = D_{c,t} - L_{c,t} - E_{c,t} - V_{c,t} .$$

### Scenarios and calibration

We applied the model to simulate scenarios for the period 1965–2100 for the five scenarios of the Shared Socio-Economic Pathways (SSPs)^13^ which are used widely for assessments of climate change^31, 46^, agriculture^22, 25, 31, 45^, biodiversity^47^, or planetary boundaries^18^. They include different plausible trajectories for population growth, demographic change and income development. To create a complete dataset for all countries over the entire period 1965–2100, we combined various datasets involving historical and future income and population. The model was run not only for the future, but also for the past to further complete data. Yet, for the historical period (1965–2010), the model was calibrated to meet historic data for body height^51^, BMI^57^, per-capita food demand and dietary composition^7^ using additive calibration values that were derived by subtracting model projections for the historical period from reported data. These factors were kept constant from 2010 onwards and added to the projected values. Calibrated values were cut off at values of below zero and share estimates were cut off above one. For BMI we used the calibration values for 1975 to simulate 1970 and 1965, for which no global BMI dataset exists.

## Supplementary information

Supplementary Information 1.

Supplementary Information 2.

## Data Availability

The code of the food demand model used for this publication as well as regular updates to the model can be downloaded and installed from Github (https://github.com/magpiemodel/magpie/releases/tag/v4.1.1). Additionally, the model has been archived via Zenodo (https://zenodo.org/record/3701289). Model outputs and analysis scripts used for this study as well as a guide for running the food demand model can be downloaded from https://zenodo.org/record/4034439. Regression analysis was performed using our R library mrregression (https://doi.org/10.5281/zenodo.3699647) and data input processing scripts of the R library moinput (https://doi.org/10.5281/zenodo.3699594).
