# Supplementary material for: The ongoing nutrition transition thwarts long-term targets for food security, public health and environmental protection
Source: Sci Rep. 2020 Nov 18;10:19778. doi: 10.1038/s41598-020-75213-3 (PMC7676250; doi:10.1038/s41598-020-75213-3)
Supplement: Supplementary file 2 — Supplementary Information 2. [file 41598_2020_75213_MOESM2_ESM.docx]

**Supplementary Information for the manuscript**

**The ongoing nutrition transition thwarts long-term targets for food security, public health and environmental protection**

**Authors**

Benjamin Leon Bodirsky*^1^, Jan Philipp Dietrich^1^, Eleonora Martinelli^1^, Antonia Stenstad^1^, Prajal Pradhan^1^, Sabine Gabrysch^1,2^, Abhijeet Mishra^1^, Isabelle Weindl^1^, Chantal Le Mouël^3^, Susanne Rolinski^1^, Lavinia Baumstark^1^, Xiaoxi Wang^4,1^, Jillian L. Waid^1^, Hermann Lotze-Campen^1,5^, Alexander Popp^1^

**Affiliations**

^1^ Potsdam Institute for Climate Impact Research, Potsdam, Germany

^2^ University of Heidelberg, Institute of Public Health, Heidelberg, Germany

^3^ INRAE, Agrocampus-Ouest, SMART-LERECO, Rennes, France

^4^ Zhejiang University, Department of Agricultural Economics and Management, Hangzhou, P.R. China

^5^ Humboldt University, Berlin, Germany

* Corresponding author. Contact: [bodirsky@pik-potsdam.de](mailto:bodirsky@pik-potsdam.de), Potsdam Institute for Climate Impact Research, P.O. Box 60 12 03, 14412 Potsdam, Germany

## : General modelling approach

### Programming Architecture

Scenarios were simulated using an open-source food demand model written in the programming language GAMS. It is an updated version of Bodirsky et al (2015)(*1*, *2*), which has been also used in FAOs outlook “Future of food and agriculture: Alternative pathways to 2050”(*3*).

Fig. S1 provides an overview of the model design and the sequence of estimations. S2 - S7 describe the individual model processes, the data preparation and the estimation of regression parameters, which have been carried out using our open-source data processing framework madrat(*4*) written in the programming language R(*5*). S9 provides a five-fold cross-validation analysis and S10 compares the results to other projections.


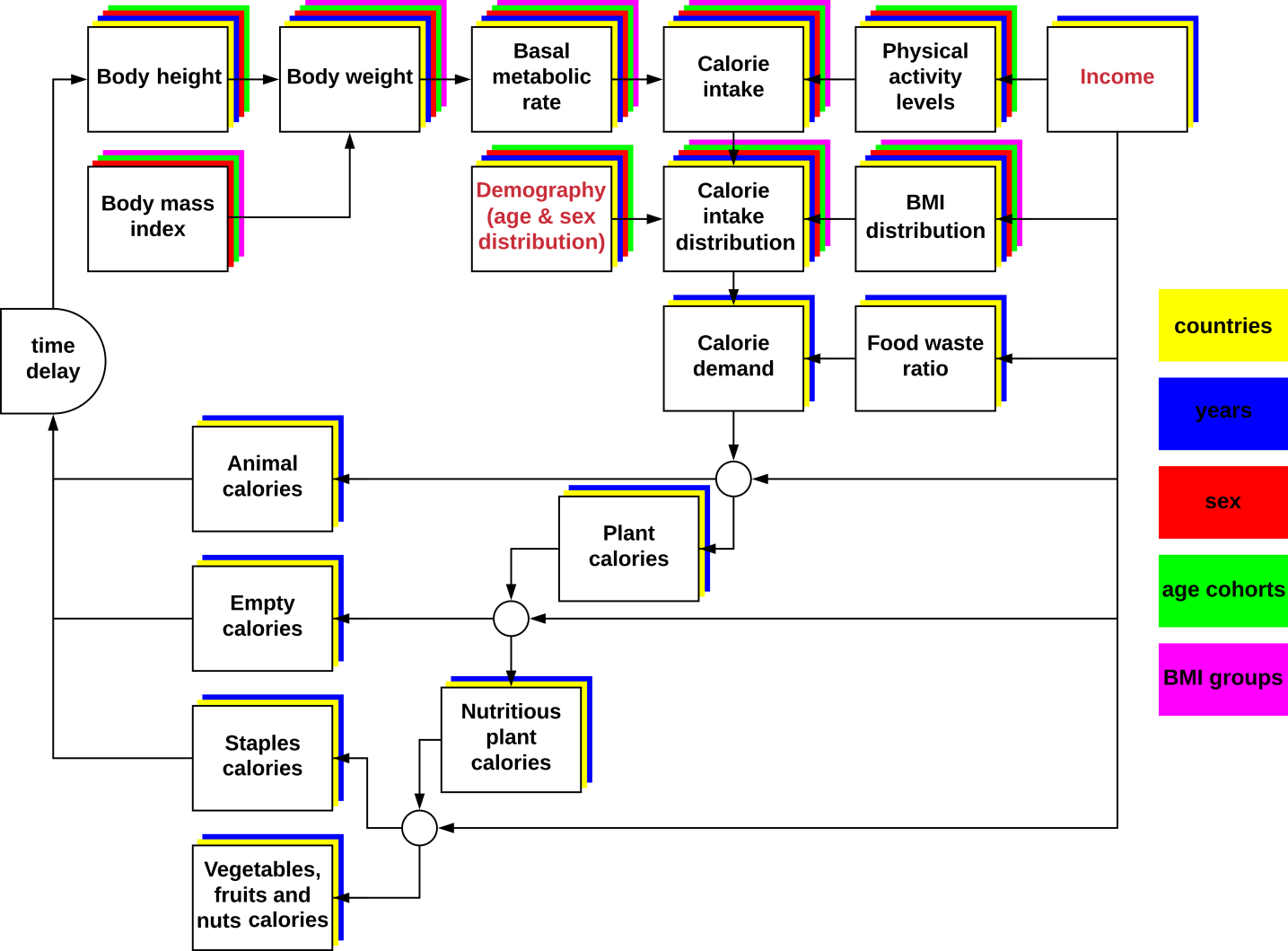


Fig. S1 | Model design. Red font indicates model drivers. Colored layers indicate the dimensionality of the variables.

The food demand model is part of the Model of Agricultural Production and its Impact on the Environment (MAgPIE), a modular open source framework for modeling global land-systems(*6*–*9*). For this study, the food demand model was run in its standalone-version, while it can be also executed in coupling with MAgPIE. The documented model code can be downloaded at <https://github.com/magpiemodel/magpie/releases/tag/v4.1.1>. Model outputs and analysis scripts used for this study as well as a guide for running the food demand model can be downloaded from <https://doi.org/10.5281/zenodo.3699647>.

### Data

Table S1 lists the data sources used for model parametrization and scenario runs.

Table S1 | Data sources for model parametrization and scenario runs. Further data for current income and population were used from (46–50).

| Datasource | Indicators | Time-period | Spatial resolution | Subpopulations |
| --- | --- | --- | --- | --- |
| FAOSTAT(2015)(*10*) | *Food demand* ( dietary energy supply in kcal per capita) | 1961-2011, yearly | 171 countries | - |
| WHO Global Health Observatory Database (2018)(*11*) | Physical inactivity adults | 2010 | 146 countries | By sex |
| WHO Global Health Observatory Database (2018)(*11*) | Physical inactivity school-going adolescents | 2010 | 120 countries | By sex |
| NCD-RisC (2017)(*12*) | BMI grouped population shares | 1975—2016, yearly | 200 countries | By sex, age, bmi-classes |
| NCD-RisC (2016)(*13*) | body height (cm) | 1914-2014, yearly | 200 countries | By sex |
| FAO/WHO/UNU (2004)(*14*) | Food energy intake | - | - | By sex, age, weight |
| FAO/WHO/UNU (2004)(*14*) | Food energy intake |  |  | By sex, age, height, weight |
| Froehle (2008)(*15*) | Energy requirements | - | - | Age, sex, weight, temperature |
| KC and Lutz (2017)(*16*) | Population | 1970—2100, 5-yearly | 194 countries | Age, sex |
| SSP-Database (2015) (*17*) | Population | 2000—2100, 5-yearly | 194 countries | - |
| SSP-Database (2015) (*17*) | Income per capita | 2000—2100, 5-yearly | 194 countries | - |
| Worldbank (2013)(*18*) | Population | 1960—2014, yearly | 212 countries | - |
| James et al (2012)(*19*) | Income per capita | 1950—2015, yearly | 210 countries | - |

### Parametrization

We used regression analysis to parametrize the model using several cross-country time-series datasets (Table S1). The data was weighted by population, and countries with less than 1 million people were excluded from the regressions due to data quality concerns. The regressions coefficients were estimated using the function nls() from the R-package stats(*5*). For each regression, we provide the number of reported data points, the Efron’s pseudo R² between reported data and projections, the standard error and the loglik value (see Extended Data). For each regression coefficient, we calculated the standard error, the t-value, and the confidence interval (see Extended Data). Regression analysis was performed using our R library mrregression (<https://doi.org/10.5281/zenodo.3699647>) and data input processing scripts of the R library moinput (<https://doi.org/10.5281/zenodo.3699594>).
We decided that all estimated parameters shall have no regional and no temporal dimension for several reasons. First, we consider our included drivers with spatial and temporal dimension (demography, anthropometry and income) to explain sufficient parts of the variance in the reported data over time and geography. Second, we did not use regionalized parameters as we want to make long-term projections under economic growth, so all countries move rapidly out of the domain for which reported data on the country level exist. Given the high economic growth expectation until 2050, this also applies for parametrization based on larger world-regions. We therefore consider it more adequate to parametrize with countries of comparable socio-economic status, rather than to extrapolate out of the domain of observed values. Thirdly, country and time-dependent parameters lack explanatory power; there is no rational that time or geography per-se would influence eating habits. Drivers should rather be included specifically. So if climate induces geographical differences by higher energy needs, climate instead of geography should be included. Similarly, it is difficult to interpret which time-trends exist that are not already covered by demographic change and by economic development; without specifying them, it is also uncertain whether they will persist or not. Instead of including time and geography into the parametrization, we instead chose to include an additive country-specific calibration values, and to discuss remaining time-trends.

## : Body height regression

Body height observations of 18-year old males and females by birth-year were obtained from the NCDRisc Consortium (*13*). In order to construct a 5-yearly age cohort dataset for all age-classes with height distribution for the whole population, we combined body height observations with a demographic dataset on age-distribution, attributing each 5yr cohort the average body height of its birth years (*20*, *21*). As the reported data on height starts in 1896, it is not sufficient to represent the height of the 85+ age cohorts in 1965, where we assumed constant height of the 1896 birth year cohort. For children (0—14), we used the WHO growth standards for girls and boys and scaled it proportionally to the height of the 15-19 year olds age-cohort.
Next to subclinical and infectious diseases, food security and child feeding practices are the major drivers of stunting (*22*). In particular, longer breast-feeding, greater dietary diversity, protein intake, and the consumption of food from animal origin are associated with higher body heights (*22*). To project body height of 18yr old adults based on an indicator for the quality of the diet, we performed regressions (Fig. S2) with the food demand for animal-based products, legumes and oils as predictor for height, which show a much higher correlation with body height than total calorie availability. To account for the growing period and to avoid circularities in the model, the body height of the age-cohort 15--19 is predicted by the demand in the preceding three 5-year time-steps, covering the time-span of approximately one year before pregnancy until the completion of the 14^th^ life year.


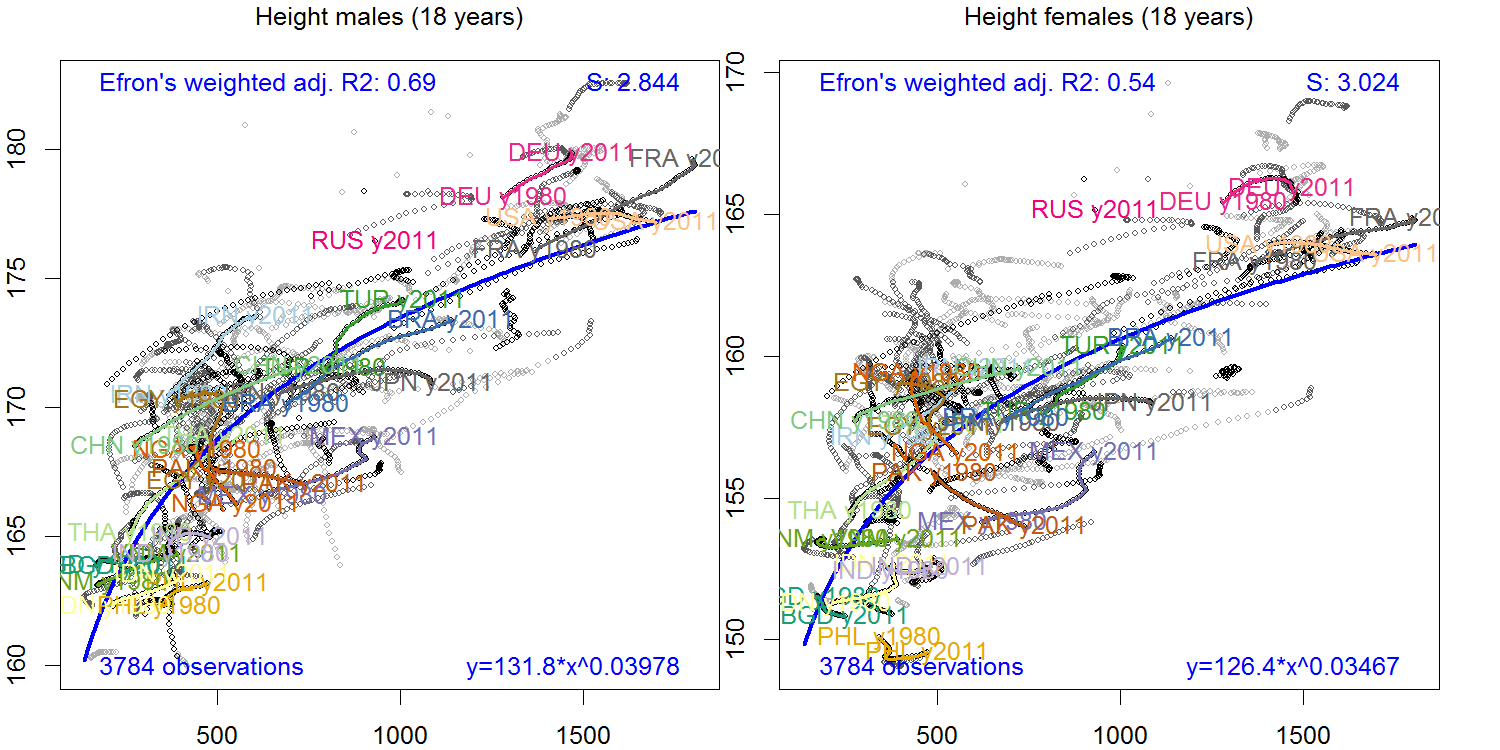


Fig. S2 | *Body height of 18 year old males and females in cm and its relationship with the average consumption of calories from animal-products, legumes and oils within the last 15 years. Points indicate reported data of a country in a year, darker points have higher population. Thick blue lines are the estimated functional relationship between relative intake levels and income. Thin coloured lines indicate the development of the 20 most population-rich countries over the observed period.*

## : Physical (in)activity

Physical activity estimates are based on a dataset on physical inactivity, which are provided by the WHO Global Health Observatory Database (*11*) for the year 2010 for 146 countries for adults and 120 countries for school-going adolescents. For the remaining countries and years, data was interpolated as follows: For children (0—14), we used the global average share of physically inactive school-going adolescents of 77.6% for males and 83.9% for females. For adults, the higher rates of manual labor in low-income countries lead to a clear pattern of more physical inactivity in high-income countries (*23*). Based on the averages in low-income and high-income countries, we therefore adopted income-dependent average proportions of physical inactivity of 27.7% for men and 37.6% for women in high-income countries (>10 000 USD05/capita), as opposed to 10.7% and 22.4% for men and women in low-income countries (<1 000 USD05). In medium-income countries we interpolated between those values. As the dataset only contains data for the year 2010, we assumed for adults that physical inactivity changed over time using the income dependency, yet calibrating the values to the reported data in 2010 using an additive term. A lower limit of 3% of inactive adults was set as there was no reported data with less than 3% inactivity of a population.
Due to higher activity of working-age population, working-age adults have approximately one third lower inactivity rates than adults in retirement age (*24*). In the absence of age-group specific data, we decreased the inactivity levels of the age-groups 20-60 and increased the inactivity levels of the 60+ population accordingly across all countries.
Finally, the share of physically inactive population was translated into average physical activity levels $A_{a,s,c,t}$ by assuming a value of 1.53 (sedentary or light activity lifestyle) for inactive and 1.76 (active or moderately active lifestyle) for the remaining population (*14*).

## : Metabolic equations

A number of different sets of equations have been proposed to estimate basal metabolic Rate (BMR) based on anthropometric characteristics like body weight, height or age. Weijs et al (*25*) lists 19 different sets of equations and validates their outcome using observational data with a special focus on underweight individuals. For this study, we use the WHO/FAO/UNU (2004)(*14*) equations (Eq3) which are based on the Schofield equations for weight, age and sex(*26*, *27*). For comparison (see S6), we also implemented another set of equations by Schofield that also include height(*26*, *27*), as well as equations by Froehle(*15*) that account for age, weight, sex and mean outdoor temperature $T_{c,t}$ (Eq 3c). We selected Eq3a the model as it provides similar results as Eq3b and Eq3c with a simpler model, and as it avoids the problems of multi-collinearity that occur when multiple explanatory variables are correlated, such as body weight and height.

$$Eq3a: I_{b,a,s,c,t}={(\gamma}_{a,s}W_{b,a,s,c,t}+\delta_{a,s})A_{a,s,c,t}$$

$$Eq3b: I_{b,a,s,c,t}={(\nu}_{a,s}W_{b,a,s,c,t}+\xi_{a,s}H_{a,s,c,t}+o)A_{a,s,c,t}$$

$$Eq3c: I_{b,a,s,c,t}={(\rho}_{a,s}W_{b,a,s,c,t}+{\varsigma T}_{c,t}+\sigma)A_{a,s,c,t}$$

## : BMI estimation

To estimate the BMI distribution within the population, we use a nested tree (Fig. S3). Each branch splits a population into different BMI ranges. For *working-age adults* (aged 15—59) and *retirement-age adults* (aged 60+) we use the BMI classes “<18.5”, “18.5—20”, “20—25”, “25—30”, “30—35” and “>35”. As healthy BMI changes during growth, we distinguish for *children* (aged 0—14) the BMI groups which diverge by “<-2”, “-2 — ‑1”, “-1 — +1”, “+1—+2” and “>+2” standard deviations from the WHO BMI recommendations(*28*, *29*). BMI <18.5 for adults and <-2SD for children are defined as underweight, BMI >25 and >+1SD as overweight and obese, and BMI>30 and >+2SD as obese.

The shares are derived either through regressions of BMI population shares (*12*) with per-capita income (Fig. S4 - Fig. S6) or as the residual adding up to 1. Fig 2 in the main manuscript displays the parametrized functions over different BMI and age groups.

| 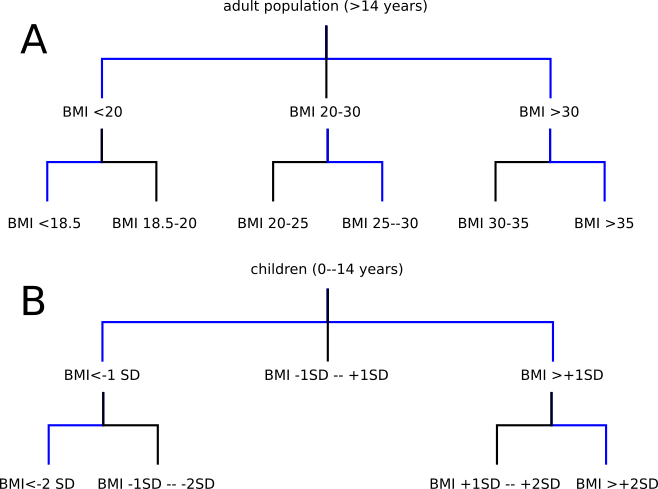  Fig. S3 \| *Nested tree used to estimate the share of populations belonging to a BMI group for adults (A) and children (B). Blue branches are estimated using regressions and black elements are the residual shares.* |  |
| --- | --- |


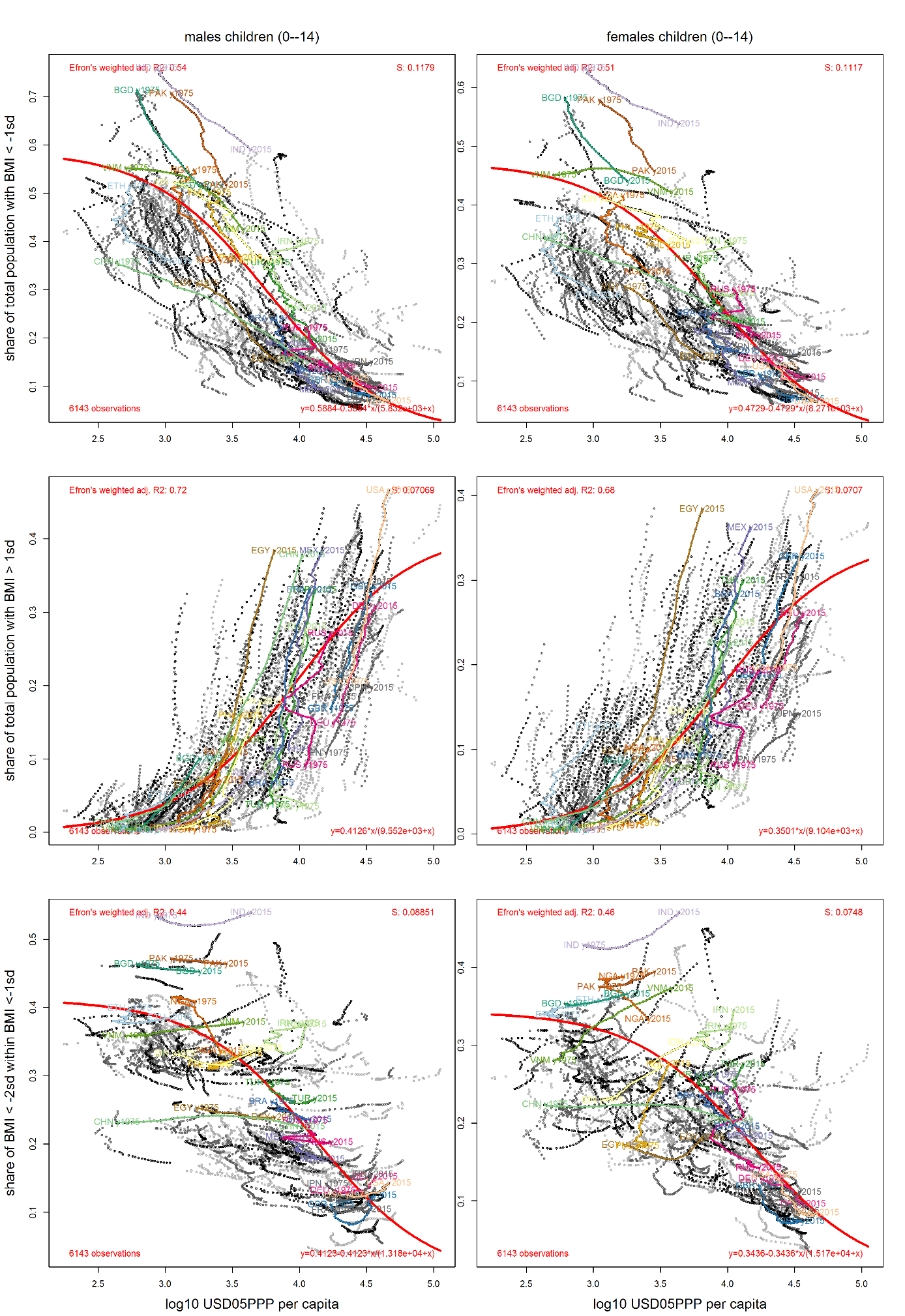


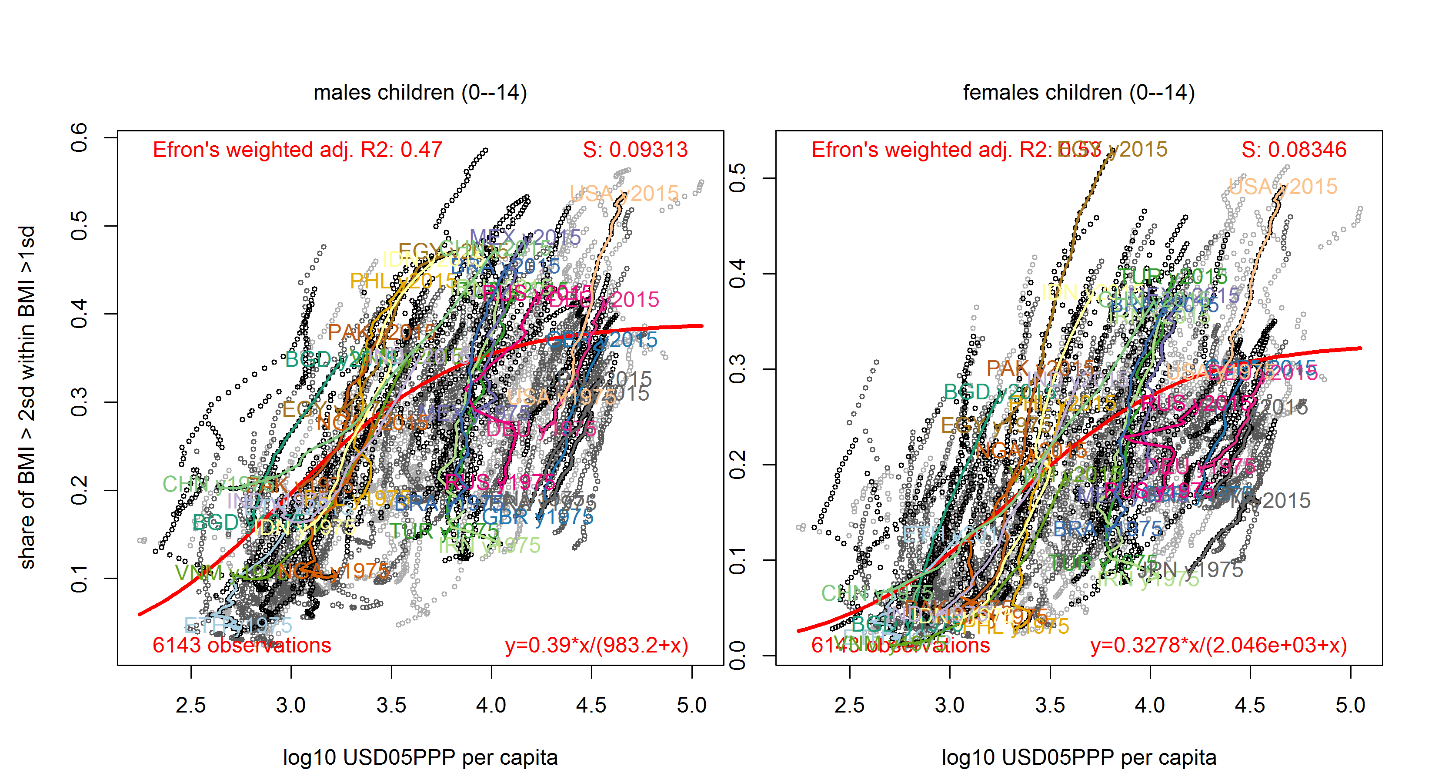


Fig. S4 | *Regressions for BMI distribution among children (0—14 years). Coefficients and statistical indicators can be found in the Extended Data. Points indicate reported data of a country in a year, darker points have higher population. Thick red lines are the estimated functional relationship between BMI population shares and income. Thin coloured lines with ISO-3-country codes indicate the development of the 20 most population-rich countries over the observed period.*


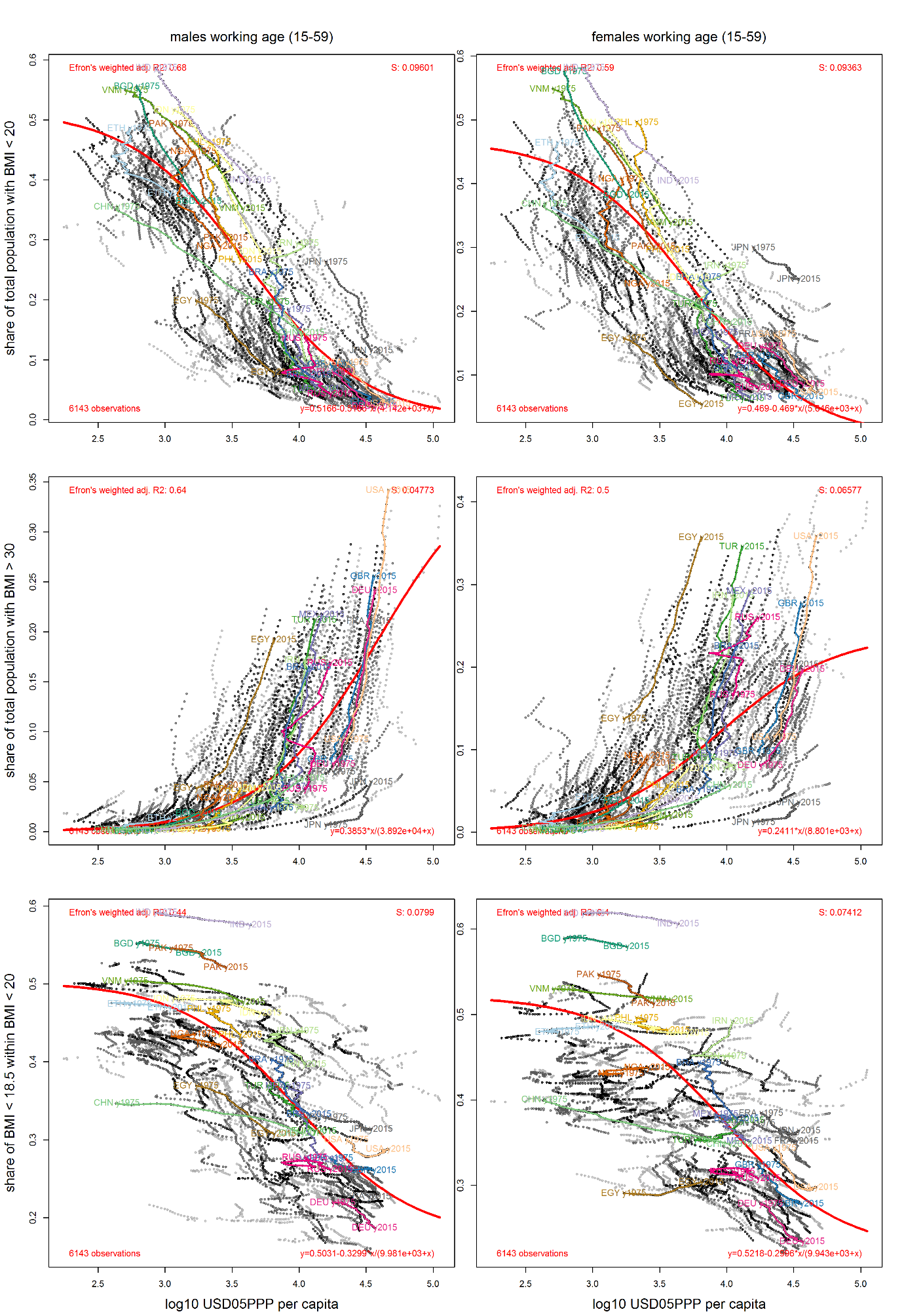


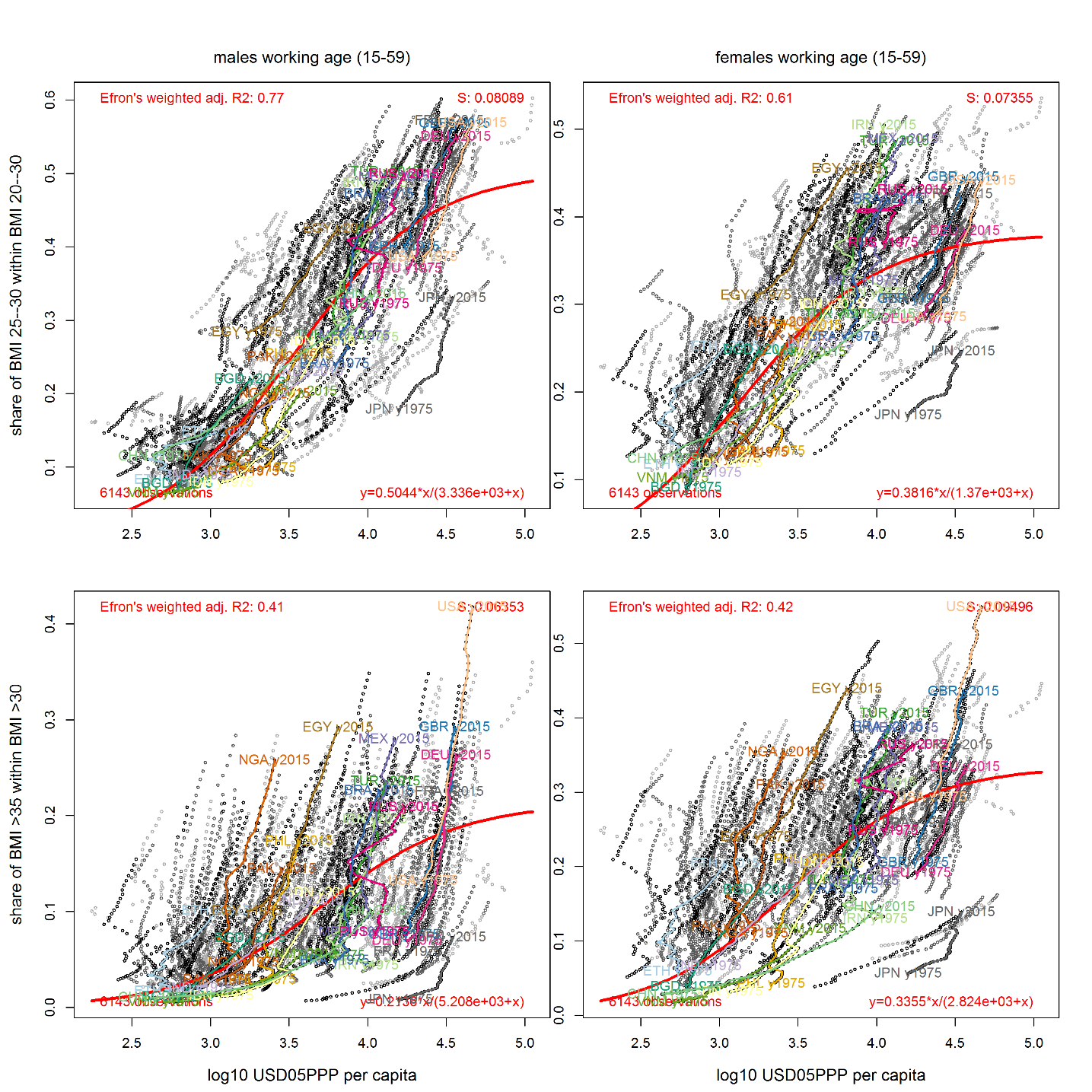


Fig. S5 | *Regressions for BMI distribution among working age adults (15—59 years). Coefficients and statistical indicators can be found in the Extended Data. Points indicate reported data of a country in a year, darker points have higher population. Thick red lines are the estimated functional relationship between BMI population shares and income. Thin coloured lines with ISO-3-country codes indicate the development of the 20 most population-rich countries over the observed period.*


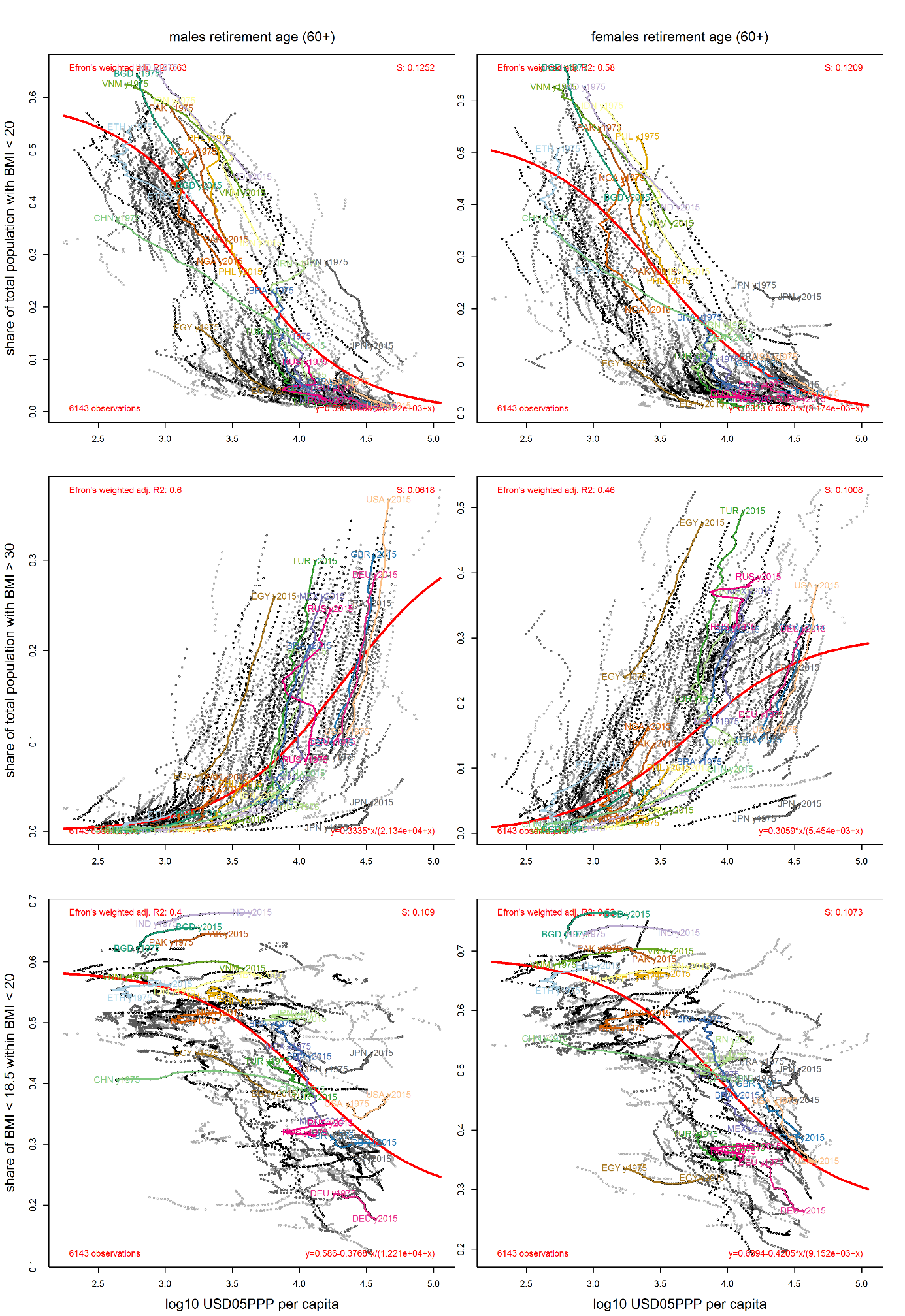


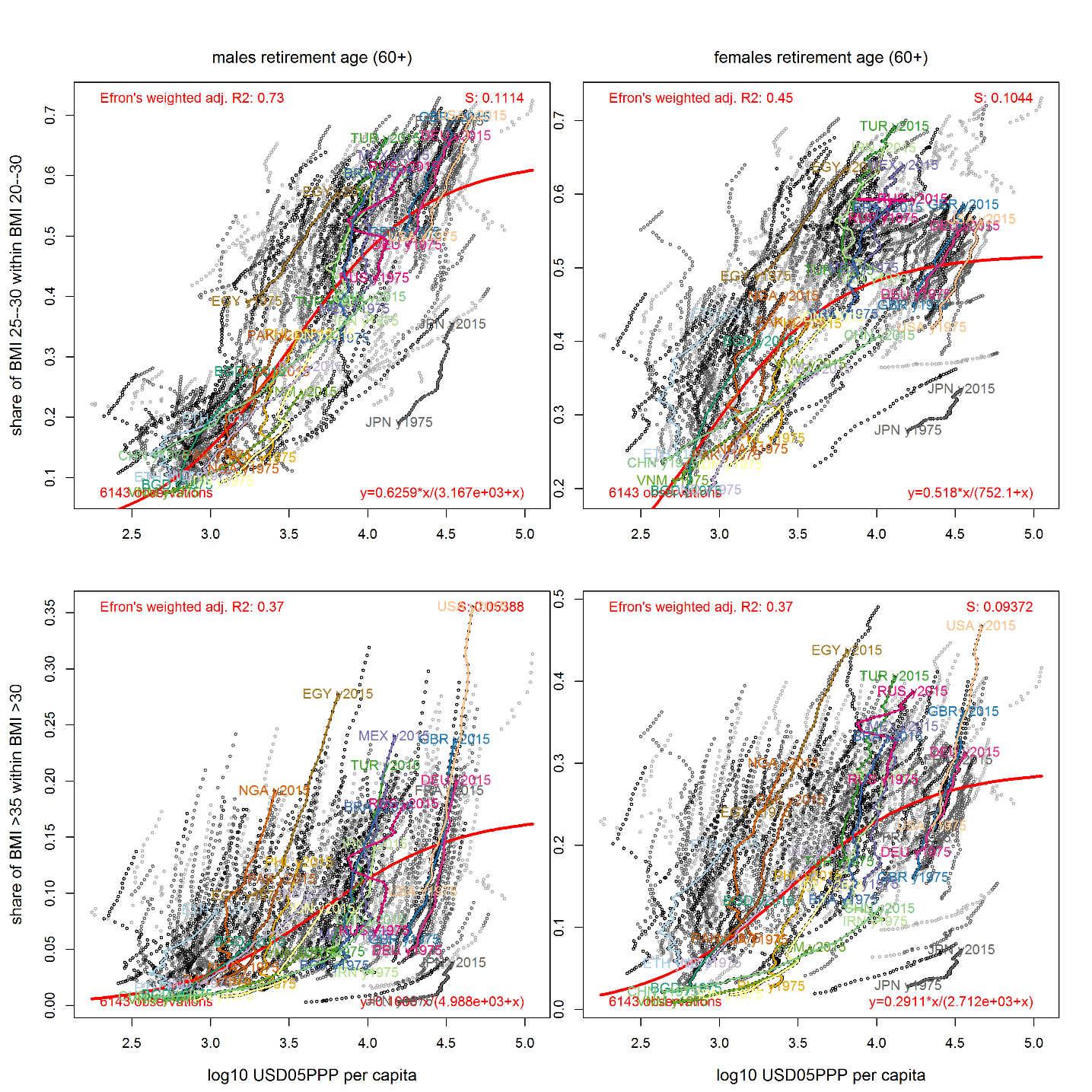


Fig. S6 | *Regressions for BMI distribution among retirement age adults (60+ years). Coefficients and statistical indicators can be found in the Extended Data. Points indicate reported data of a country in a year, darker points have higher population. Thick red lines are the estimated functional relationship between BMI population shares and income. Thin coloured lines with ISO-3-country codes indicate the development of the 20 most population-rich countries over the observed period.*

## : Food waste ratio

Food demand, defined as the food calorie availability by the FAO in kcal/capita/day (*10*), is the average quantity of calories that is available to consumers, including purchased food but also self-cultivation and out-of-house consumption, but excludes feed, material usage or losses within the food supply chain. Besides food actually being eaten (food intake, see section S4) it also includes food waste after wholesale marketing and within households, which can be a considerable share of food demand (*23*, *30*).

We estimate the food waste ratio, defined as the ratio between food demand and intake, using a regression with per-capita GDP in USD05PPP/capita (*19*) (Fig S7). We use a food waste ratio instead of an additive term, because we expect that food waste is not only dependent on income, but also on the quantity of intake, as higher intake also requries higher storage and preparation losses. We use a Michaelis Menten functional form, because we expect that food demand will converge to food intake for very low incomes and that the the food waste ratio will saturate for very high incomes.

The food waste ratio should by definition be larger than one. There are however a number of countries where estimated intake is higher than FAOSTAT food availability, including most importantly China, Nigeria and Vietnam during some time-period. We investigated different reasons for this mismatch. The lower energy intake of underweight individuals are already considered in our estimates, which however assume a state of body weight equilibrium. Ongoing weight-loss during starvation mode could explain temporary overestimation of intake (*31*), but is an unlikely explanation of intake being lower than demand over several years or even decades as observed in our data. Also an overestimation of physical activity levels is an unlikely explanation given that our assumptions of sedentary to moderate activity are likely at the lower end expected for field-labour intensive work in developing countries (*23*). According to Schofield (1985), the method may overestimate BMR in countries like India, while it may underestimate the intake in Northern Europe or America, even if individuals have the same bodyweight. Whether ethnicity may play a role for metabolic requirements is still unclear (*14*) with studies coming to different conclusions (*32*, *33*), and differences may also depend on early life exposure to suboptimal nutritional environments (*14*). Differences in outdoor temperature may be one explanation for this (*26*), but using a set of metabolic equations that accounts for temperature differences (*15*) did not remove the mismatch (Fig S7c) while it even used different datasets for the regression. Finally, national dietary surveys are often inconsistent with FAOSTAT estimates (*34*, *35*). While FAOSTAT is supposed to include also subsistence and backyard farming, data quality in developing countries is likely poor (*23*) and may underestimate these activities (*34*).


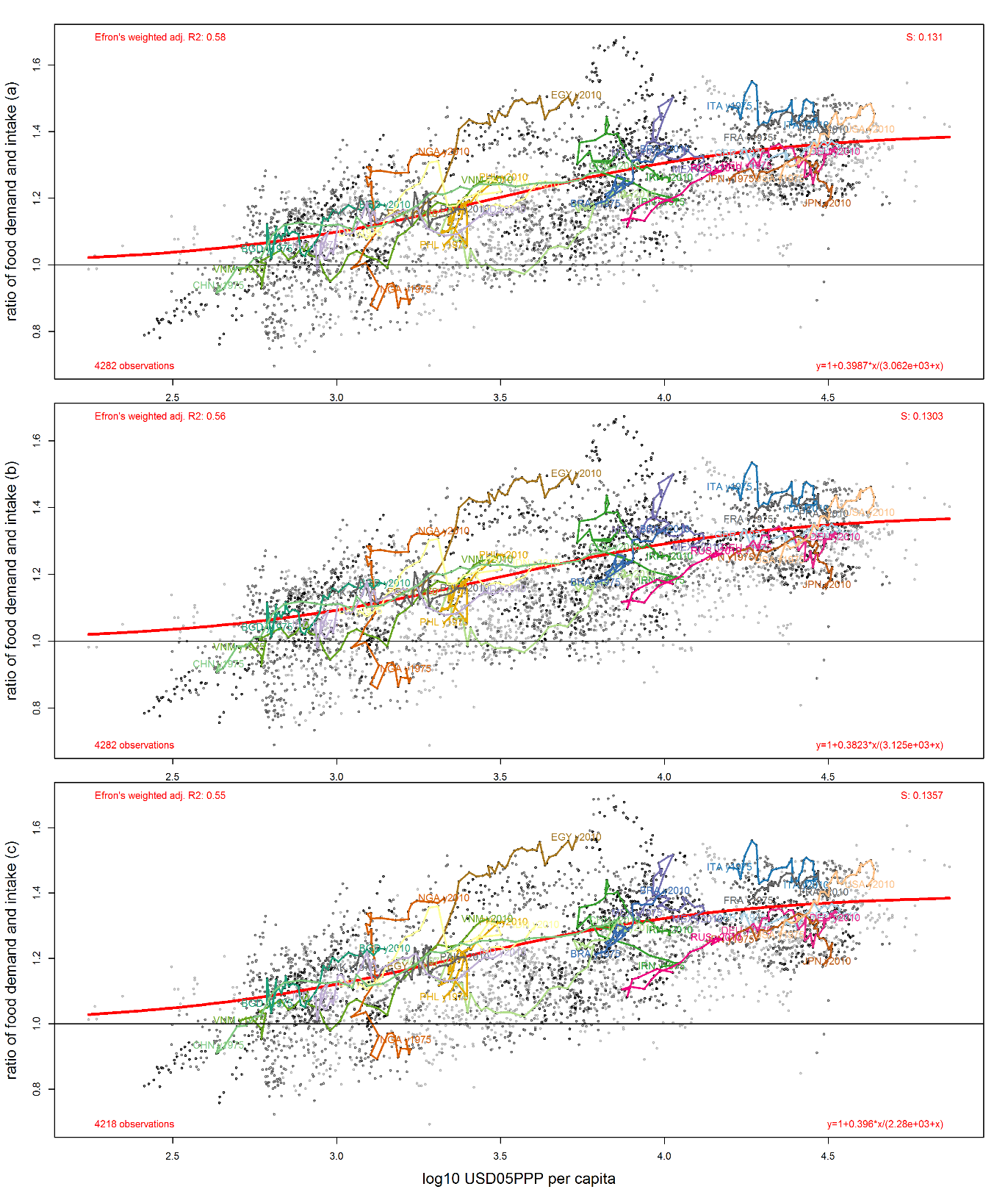


Fig S7 | *The ratio of food demand and intake, plotted against log10 per-capita income. The ratio is sometimes below one, indicating inconsistencies in data. Here we compare the ratio under three different models to estimate intake: (a) Schofield body weight according to eq. 3a, (b) Schofield body weight and height according to eq. 3b, and (c) Froehle et al* (*15*) *with bodyweight and temperature according to eq. 3c. Points indicate reported data of a country in a year, darker points have higher population. Thick red lines are the estimated functional relationship between BMI population shares and income. Thin coloured lines with ISO-3-country codes indicate the development of the 20 most population-rich countries over the observed period.*

## : Dietary composition

We subdivide food demand into “animal source foods”, “empty calories”, “fruits, vegetables & nuts” and “staples” using a nested tree (Fig. S1). These categories were selected because they are nutritionally important macro-groups, and also strongly overlap with agro-economic sub-sectors relevant for land-system modelling. Within those food groups, we found no clear income dependency that would allow further differentiation into more detailed food groups. In our model, we therefore keep the relative shares of products within each food group constant. This influences the model projections only for the body height projections, where we include oils and pulses next to the animal source foods. Due to their importance as starchy staples in tropical countries, plantains and bananas were attributed to staples and not to fruits, vegetables & nuts. We used calorie shares, as food weight strongly varies with water content of different product types, and as vegetables, fruits, sugar and oil have little or no protein. The functions to estimate the dietary energy shares were parametrized using a regression analysis between FAOSTAT dietary energy supply(*10*) and per-capita income(*19*) (Fig. S8).


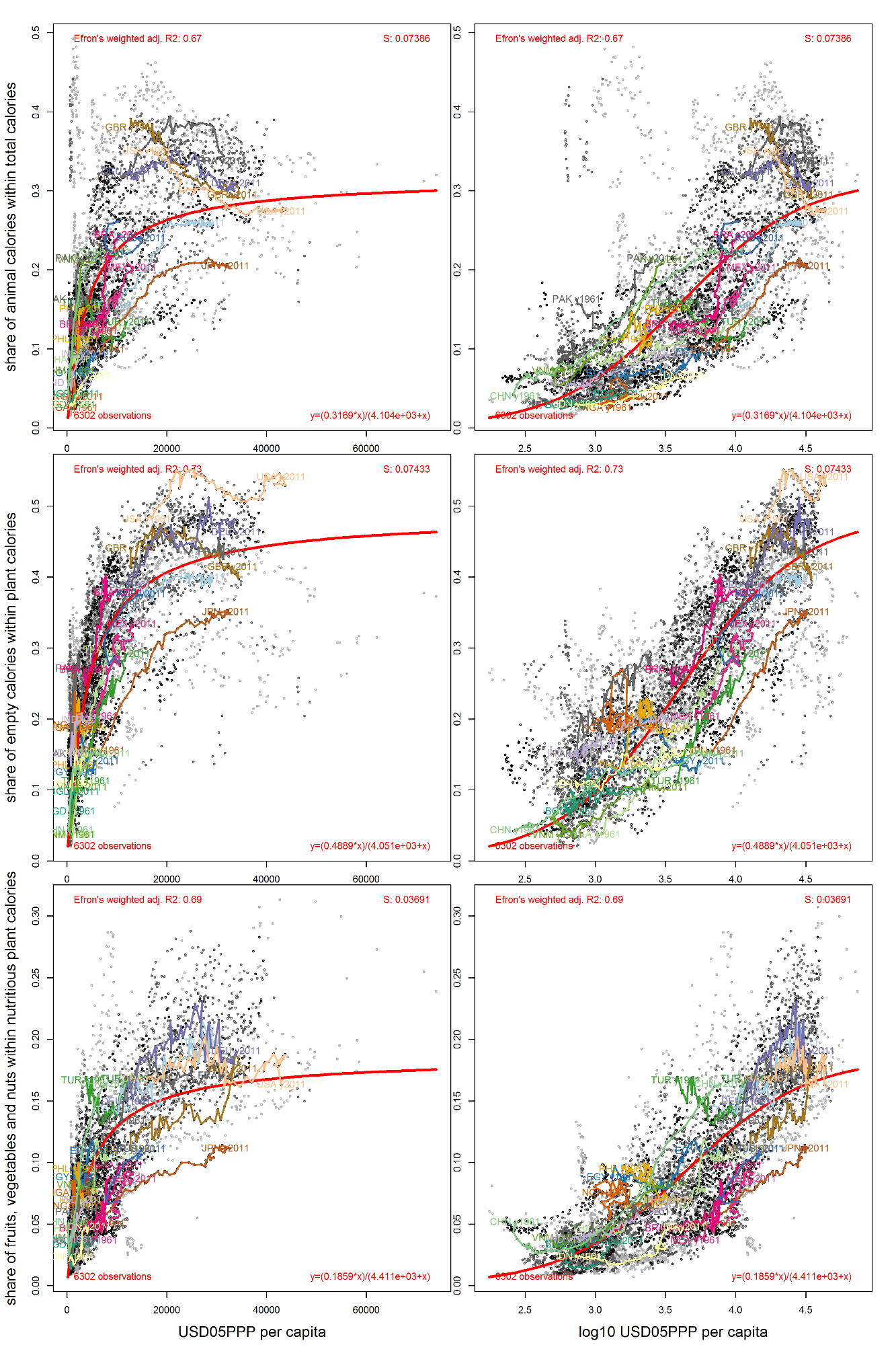


Fig. S8 | *The relationship between dietary composition and income. Left plots show per-capita income on the x-axis, right plots log10 per-capita income for better presentation of low-income countries. The first row is the share of animal-based calories in the total calorie demand, the second row is the demand for the sum of sugar, oils and alcohol as a share of non-animal calories, and the third row the demand for fruits, vegetables and nuts as a share of the calories which are not animal-based and not from oils, sugar or alcohol. Thin lines show the development of the 20 most population-rich countries, accounting for roughly two thirds of the world population in the course of the century. Each point indicates reported data of a country in a year, darker points have higher population. Thick red lines are the estimated functional relationship between dietary composition shares and income.*

## : Completion of missing data for scenario projections

We decided that for the estimation of scenarios, a complete coverage of the world population is of higher importance than high data accuracy, in particular if the scenarios shall be used to estimate environmental pressure from the food system. So instead of excluding countries with incomplete data, we assembled data for all 249 countries or territories with ISO-3 country codes.
Worldbank provides historic population data for 212 countries and territories, and James et al (2012)(*19*) provides per-capita income data for 210 countries and territories. SSP projections(*17*) include 194 countries. We completed the data for other minor countries and territories with various sources (*36*–*40*). When time series were not available, numbers were kept constant. Demographic structure data(*16*) has only 193 countries. For remaining countries we applied the demographic structure of a country of the same world region with a similar economic development state. BMI shares were completed using our income regression model. Body height values of 18yr old adults was completed with world averages since 1914, as the regression could not be applied for the whole time period. As WDI and SSP data do not completely match for 2010, we converged the dataset between 2010 and 2020. We also had to adjust for the transition of countries. In case a country split in two, we also split the historical values (in case of absolute numbers we used population as weight, in case of relative numbers we assumed the same value for both parts of the country). In case of a unification, we aggregated the values (by sum or population-weighted average), such that our dataset expresses all countries in countries existing in the year 2010, even if for parts of the history they belonged to different states which ceded to exist.

We used no self-completed data for the parametrization of the regression analysis, with the exception the food waste ratio. Here, the scarce PAL data would have strongly reduced the number of observations. As PAL values only vary between 1.53 and 1.76 the inclusion of the completed data will not have major effects on the estimates of intake, which are more determined by demographic and anthropometric structure.

## : Out-of-sample testing of the model

Out-of-sample testing can be used to evaluate how reliable a predictive model can perform in practice, and is a valuable tool in particular to identify over-fitted models.
Here, we perform a five-fold cross-validation of our entire model. All tuples of country-year combinations in the reported data were randomly assigned to five groups. Then, all regressions were estimated five times, each leaving out rotation-wise one fifth of the tuples as test data. Consequently, the model was run five times with the five sets of parametrizations (documented in the Extended Data along with the parametrization of the default model). The projections of all five model runs were merged to a single dataset in a way that each model contributed the projections for the tuples that were not used for its parameterization. We tested the combined dataset for the following indicators: food demand (kcal/capita/day), animal source food (kcal/capita/day), empty calories (kcal/capita/day), fruits, vegetables and nuts (kcal/capita/day), obesity (population share), overweight (population share), and underweight (population share). As each of the tested indicators is the outcome of the combination of different functions that were estimated independently, we thereby do not only measure the fit of individual regressions, but also their interplay, evaluating a part of the structural uncertainty of our model.
First, we compare the combined results of the five-fold cross-validation models to the results of our default model. We find that the model results remain robust to the omission of data (Table S2), with an coefficient of determination (R²) > 0.99 between the results of both models, and low values for Root Mean Standard Deviation (RMSD), and Median Absolute Deviation (MAD). This indicates that our model is not overfitted.

Table S2 | Coefficients of determination (R²), Root Mean Standard Deviation (RMSD), and Median Absolute Deviation (MAD) between model simulations estimated with the complete data and model-simulations parametrized with the five-fold cross-validation method. Both simulations were performed with uncalibrated data, and coefficients were derived using population size of each country as weight.

|  |  | | Food demand | Animal source food demand | Empty calorie demand | Vegetables, fruits & nuts demand | Underweight | Overweight | Obesity |
| --- | --- | --- | --- | --- | --- | --- | --- | --- | --- |
|  |  |  | Kcal capita^-1^ day^-1^ | | | | Share of population | | |
| Coefficients of Determination (R²) | Not calibrated | 1965-2015 | >0,99 | >0,99 | >0,99 | >0,99 | >0,99 | >0,99 | >0,99 |
| Root Mean Standard Deviation (RMSD) |  |  | 6 | 4 | 7 | 2 | 0.001 | 0.002 | 0.002 |
| Median Absolute Deviation (MAD) |  |  | 3 | 2 | 6 | 2 | 0.001 | 0.001 | 0.001 |

Second, we compare the combined results of our five-fold cross-validation models with the out-of-sample testing data. To analyze the impact of the calibration procedure on the model output, we included two output datasets: One where the calibration was deactivated and one where the model was calibrated to the year 1975, the first year with a complete set of reported data. For all subsequent years, the calibration was fixed in the same way as the calibration is fixed to 2010 in the default projections.
We obtain for all indicators a R² > 0.675 in the model calibrated to 1975 (Table S3). The calibration improves the R², RMSD and MAD of the projections for most of the eight analyzed indicators. The calibration allows to project in particular the near-term estimates more accurately (the Extended Data provides R², RMSD and MAD for each time step), but also substantially improves the accuracy for most long-terms projections of the last observed years, 2010 and 2015.

Table S3 | Coefficients of determination (R²), Root Mean Standard Deviation (RMSD), and Median Absolute Deviation (MAD) between simulations and out-of-sample selection of reported data based on a five-fold cross validation. Coefficients were derived using population size of each country as weight. Simulations were performed with a non-calibrated model for the period 1965-2010, and with a model calibrated to the first year with full a full set of reported data (1975) for the period 1980-2010. Time periods differ by indicator due to different availability of reported data. Further detailed results by year can be found in the Extended Data.

|  |  | | Food demand | Animal source food demand | Empty calorie demand | Vegetables, fruits & nuts demand | Underweight | Overweight | Obesity |
| --- | --- | --- | --- | --- | --- | --- | --- | --- | --- |
|  |  |  | Kcal capita^-1^ day^-1^ | | | | Share of population | | |
| Coefficients of Determination (R²) | Not calibrated | 1965-2010 | 0.791 | 0.767 | 0.779 | 0.515 |  |  |  |
|  |  | 1975-2015 |  |  |  |  | 0.428 | 0.746 | 0.593 |
|  |  | 2010 | 0.712 | 0.722 | 0.561 | 0.378 | 0.418 | 0.669 | 0.560 |
|  |  | 2015 |  |  |  |  | 0.362 | 0.611 | 0.497 |
|  | Calibrated to 1975 | 1980-2010 | 0.675 | 0.897 | 0.807 | 0.767 |  |  |  |
|  |  | 1980-2015 |  |  |  |  | 0.938 | 0.823 | 0.740 |
|  |  | 2010 | 0.542 | 0.822 | 0.583 | 0.611 | 0.897 | 0.691 | 0.641 |
|  |  | 2015 |  |  |  |  | 0.891 | 0.638 | 0.556 |
| Root Mean Standard Deviation (RMSD) | Not calibrated | 1965-2010 | 241 | 156 | 156 | 59 |  |  |  |
|  |  | 1975-2015 |  |  |  |  | 0.076 | 0.082 | 0.047 |
|  |  | 2010 | 231 | 162 | 228 | 82 | 0.072 | 0.097 | 0.058 |
|  |  | 2015 |  |  |  |  | 0.072 | 0.107 | 0.068 |
|  | Calibrated to 1975 | 1980-2010 | 309 | 106 | 142 | 45 |  |  |  |
|  |  | 1980-2015 |  |  |  |  | 0.028 | 0.080 | 0.048 |
|  |  | 2010 | 342 | 138 | 227 | 72 | 0.035 | 0.104 | 0.064 |
|  |  | 2015 |  |  |  |  | 0.034 | 0.116 | 0.076 |
| Median Absolute Deviation (MAD) | Not calibrated | 1965-2010 | 155 | 95 | 101 | 29 |  |  |  |
|  |  | 1975-2015 |  |  |  |  | 0.044 | 0.044 | 0.018 |
|  |  | 2010 | 248 | 69 | 187 | 32 | 0.022 | 0.063 | 0.024 |
|  |  | 2015 |  |  |  |  | 0.027 | 0.079 | 0.032 |
|  | Calibrated to 1975 | 1980-2010 | 171 | 51 | 56 | 18 |  |  |  |
|  |  | 1980-2015 |  |  |  |  | 0.013 | 0.041 | 0.014 |
|  |  | 2010 | 124 | 106 | 105 | 47 | 0.028 | 0.073 | 0.029 |
|  |  | 2015 |  |  |  |  | 0.020 | 0.060 | 0.024 |

## : Comparison with other demand projections

Here, we compare the projections of our model to projections of other studies and discuss briefly the differences. Not all indicators have been simulated yet by other models, and not all models provide their results openly and in high resolution, so we can only compare a selection of indicators.

In our previous version of demand projections, Bodirsky et al (2015)(*1*), we used a purely statistical approach to estimate future per-capita demand and livestock demand. Two sets of functional forms were used, both dependent on income and time.


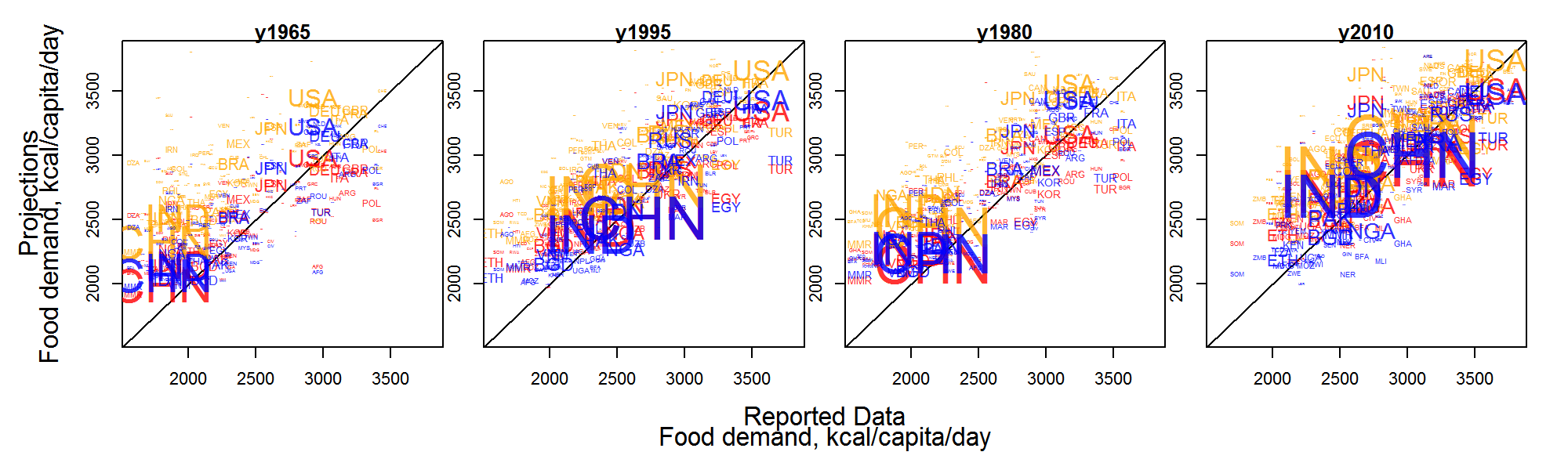


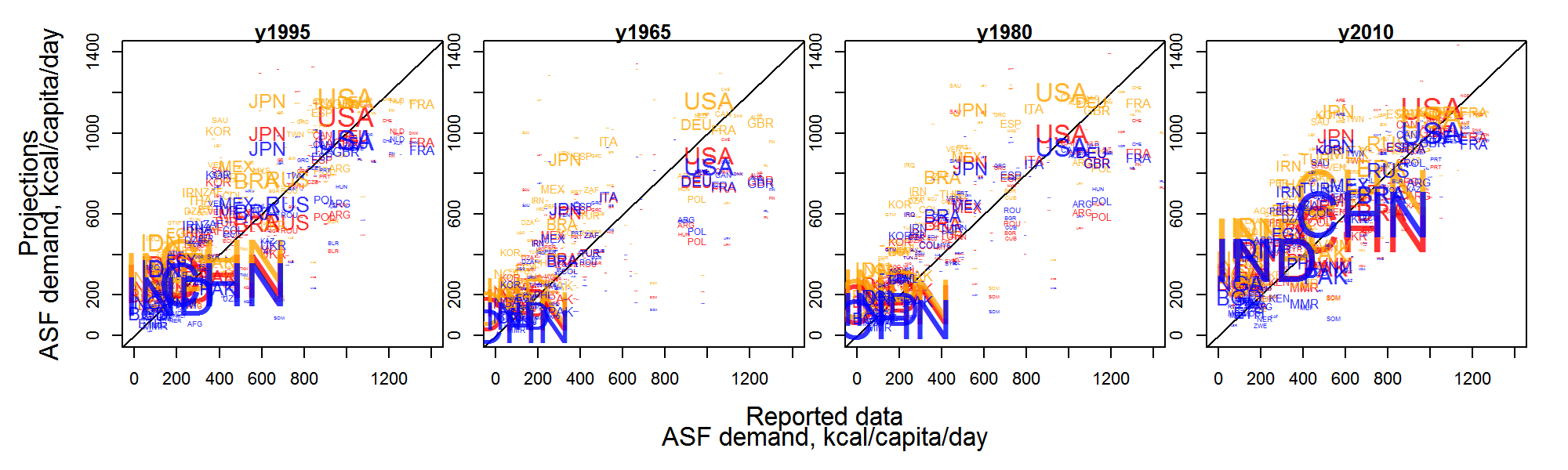


Fig S9 | Comparison of model projections for per-capita total food demand and animal-source food (ASF) demand with reported data for the model projections of this study (blue), Bodirsky et al (2015) A (red) and Bodirsky et al (2015) B (yellow). The area of the ISO-3-country code is proportional to the population of the country.

Fig S9 compares our uncalibrated estimates for per-capita food demand and animal source food demand for the years 1965, 1980, 1995 and 2010 to the results of these two models. Over all time-steps (1965-2010), our model matches reported data better than both models from Bodirsky et al (2015), with a higher R² of projected and reported values, a lower root mean square deviation and median absolute deviation (Table S4). This shows that the new model is able to replace the time-trends in the model, which are difficult to base in theory, by our additional dynamics of anthropometric change.

Table S4 | Comparison of model accuracy for this study and the two models of Bodirsky et al (2015). Indicators were estimated by comparing uncalibrated model results of all models with reported data.

|  |  | Coefficient of determination (population weighted) | Root mean square deviation  (population weighted) | Median absolute deviation  (population weighted) |
| --- | --- | --- | --- | --- |
|  |  | (R²) | (RMSD) | (MAD) |
| Food Demand (kcal/capita/day) | This study | 0.793 | 240 | 153 |
|  | Bodirsky et al (2015) A | 0.768 | 260 | 178 |
|  | Bodirsky et al (2015) B | 0.754 | 421 | 375 |
| Animal Source Food Demand (kcal/capita/day) | This study | 0.768 | 156 | 96 |
|  | Bodirsky et al (2015) A | 0.756 | 162 | 113 |
|  | Bodirsky et al (2015) B | 0.753 | 219 | 136 |

The change has implications for the projections, which is shown in Fig S10. The old projections for food demand reach maximum estimates of almost 4500 kcal/capita/day when the positive time-trends are continued (model A) and 4000 kcal/capita/day when the time-trend is faded out (model B). In contrast, the model described in this manuscript saturates before 3700 kca/capita/day. The underlying dynamics for this discontinued trend is most importantly the declining growth in calorie requirements for an ageing population (see Fig 5A, main manuscript). For animal source foods, our model simulates similar animal calories for the year 2050, but higher consumption for the year 2100, in particular when compared to model B of Bodirsky et al, which attempts to capture a declining consumption of ASF which has been observed in some high-income countries.


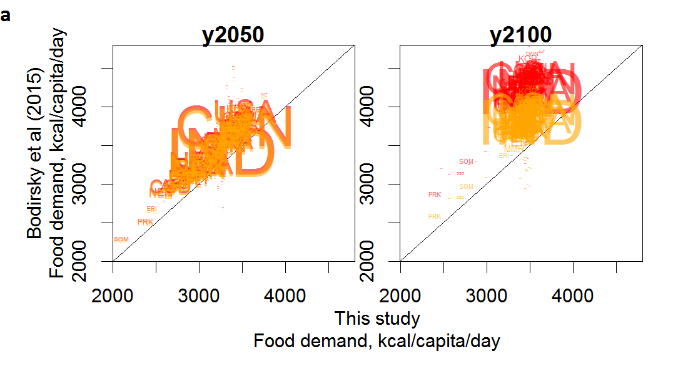

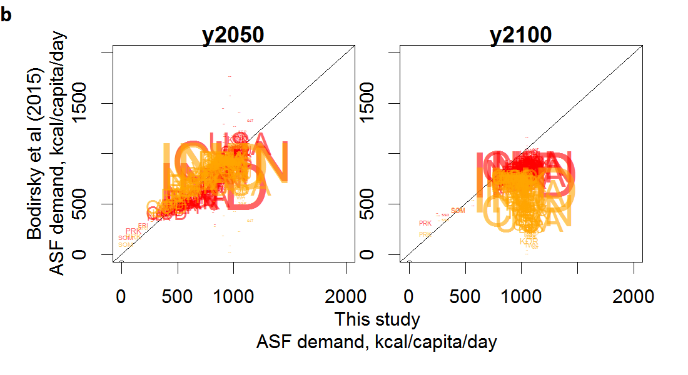


Fig S10 | Comparison of future projections for per-capita food demand (a) and per-capita demand for animal-source foods (b) of Bodirsky et al (2015) model A (red) and model B (yellow) with projections of this study. The area of the ISO-3-country code is proportional to the population of the country.

Fig. S11 compares our results to estimates to the FAO’s estimates in the World Agriculture in 2030/2050 report (*41*), and to scenarios by Bijl et al. (2017) (*42*). FAO’s study uses different population and GDP projections than those used in SSP2. Bijl et al. (2017) made estimates both using SSP2 income projections and FAO’s income projections, which helps to explain parts of the differences of the projections. Fig. S11a compares total per-capita calories for different world regions, and shows very similar results except for East Asia, where our projections are closer to FAO than to Bijl et al. (2017). Fig. S11b compares product specfific demand for developing countries. Here our projections estimate a higher increase in animal-source foods and sugar that the other models, and a higher consumption of other foods (including vegetables, fruits and nuts) than FAO, while we expect lower increase in the consumption of cereals and starchy roots and tubers.

An overview on 25 food demand estimates is given by Le Mouel and Forslund (2017)(*43*), which come to esimates of +50% to +69% for the period 2010 – 2050 for middle-of-the-road scenarios, and to +54 to +91% for scenarios with higher population or alternative diets.


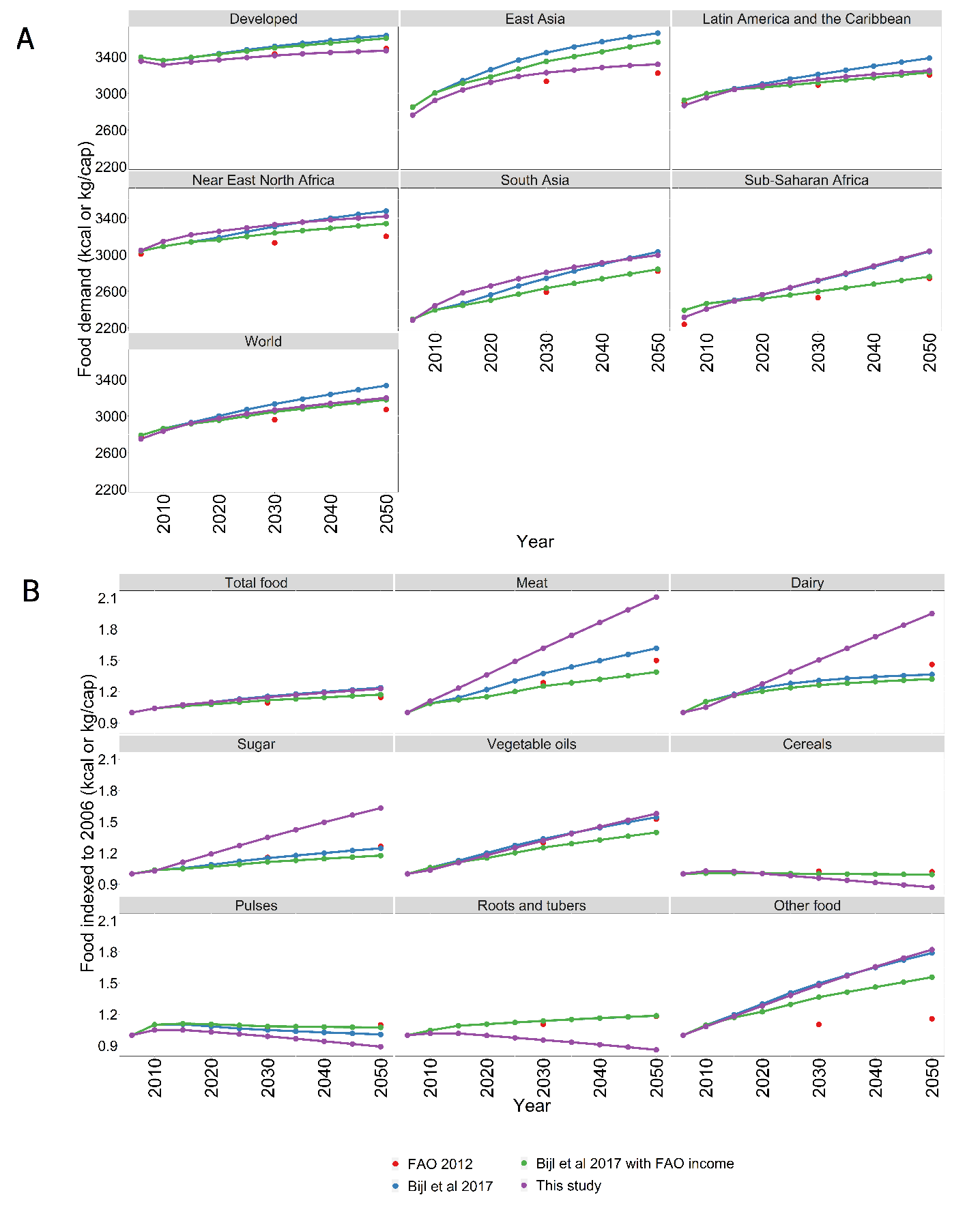
Fig. S11 | Comparison of our food demand projections with estimates by FAO(*41*) and Bijl et al.(*42*) . (A) compares total per-capita demand in different world regions, (B) compares product-specific demand in developing countries. As our projections are 5-year time-steps, we indexed our results to 2005, while the other scenarios are indexed to 2006.

## : Kaya decomposition

Fig 5 in the main text attributes the growth of per-capita demand and total animal-source food demand to the growth of different underlying drivers. The method of this Kaya-like decomposition is described in Huber et al(*44*) and assumes a multiplicative relationship of the drivers.

## : Environmental impacts of food demand

Table S5 | *Environmental impacts of food demand Findings of studies which analyze the impact of the food system on multiple environmental indicators, and which investigate measures on producer-side or consumer-side measures to reduce pollution levels.*

|  | **Environmental impacts under Business as Usual** | **Producer-side measures to reduce environmental impacts** | **Consumer-Side measures to reduce environmental impacts** |
| --- | --- | --- | --- |
| Springmann et al. (2018)(*45*) | Increase in greenhouse gas emissions, cropland expansion, bluewater use, nitrogen and phosphorus application by >50% in 2050 relative to 2010. Violation of all five planetary boundaries. | Planetary boundaries for greenhouse gases, cropland expansion, nitrogen application, and bluewater use are still violated even under ambitious technical mitigation. | Only under a combination of technical mitigation and ambitious dietary change towards a flexitarian diet and food waste reduction, the food system can function within planetary boundaries |
| Conijn et al. (2018)(*46*) | Violation of land, nitrogen, phosphorus and climate boundaries. | Technical measures insufficient to return into boundaries. | Neither demand-side nor supply-side measures alone are sufficient to protect the planetary environment. Even under a combination, the N losses may violate the planetary boundaries. |
| Gerten et al. (2020)(*47*) | In the current food system, only 3.3 billion people can be nourished within planetary boundaries of water, nitrogen, biodiversity and land. | Technical mitigation could increase the potential to 7.9 billion. | With diet change and reduced food loss, the potential could be further increased to 10.4 billion. |
| Bodirsky et al. (2014)(*48*) | Increase of nitrogen pollution by 2-56% until 2050, environmental thresholds for air, water and atmospheric pollution are violated. | Producer-side measures insufficient to return into planetary boundaries | Under a combination of technical mitigation, food waste reduction and shift towards plant-based foods, environmental thresholds may not be violated, but risk of violation still persists. |
| Henry et al. (2018)(*49*) | Most model simulations violate the planetary boundary for land until 2050. | Under high yield growth, the land planetary boundary can be met, but very low potential for bioenergy cultivation remains. | High bioenergy cultivation without violating food production and land boundary only possible under dietary change and high technological progress. |
| Kahiluoto et al. (2014)(*50*) | Planetary boundaries for nitrogen and phosphorus are violated. |  | Food waste reduction and vegetarian diets can reduce the transgression of planetary boundaries. Dietary change has to be part of the required transformation to meet planetary boundaries. |
| Weindl et al. 2017a,  Weindl et al. 2017b | Water withdrawals for irrigation will increase by around 30%, deteriorating water scarcity in many world regions. Global pasture area is stable while cropland is projected to increase, resulting in further deforestation and carbon emissions. | Intensification of livestock production systems is a driver of cropland expansion, but decreases required pasture area. Overall effects of moderate intensification processes are favorable w.r.t deforestation and carbon emissions, but detrimental for fresh water use in agriculture. | Dietary changes substantially abate deforestation, carbon emissions and green water consumption, whereas direct positive effects on blue water consumption and water withdrawals are small. |
| Stevanovic et al. 2017 (*51*) | Greenhouse gas emissions from the food system remain approximately constant until 2050 | Producer-side mitigation can reduce emissions rapidly, but at the cost of rising food prices | Consumer-side mitigation reduces both emissions and food prices. Under combined producer- and consumer-side mitigation, mitigation potential is largest and food prices only increase moderately. |
| Stehfest et al. (2009)(*52*) | Further increase of cropland cultivation and greenhouse gas emissions | A 450 ppm stabilization target is possible also without dietary change, but requires compensation of agricultural emissions by reductions in other sectors | Dietary change towards plant-based products alone is insufficient to reach 450ppm, but if combined with other measures, dietary change reduces all-sector mitigation costs by 50%. |
| Smith et al. (2013)(*53*) |  | Producer-side mitigation may have both synergies and trade-offs with other environmental targets and food security. | Diet change and waste reduction have a larger mitigation potential than producer-side mitigation, while having positive synergies with food security and other environmental targets. Combination of both producer-side and demand side measures required. |

## References

1. B. L. Bodirsky, S. Rolinski, A. Biewald, I. Weindl, A. Popp, H. Lotze-Campen, Global Food Demand Scenarios for the 21st Century. *PLoS ONE*. **10**, e0139201 (2015).

2. S. Rolinski, B. Bodirsky, “A new approach for estimating food calorie demand” (Project report for the FAO, 2015).

3. FAO, *Future of food and agriculture 2018: alternative pathways to 2050.* (FAO, Rome, Italy, 2018).

4. J.-P. Dietrich, L. Baumstark, A. Giannousakis, *May All Data be Reproducible and Transparent (madrat)* (Potsdam Institute for Climate Impact Research, Potsdam, Germany, 2018; https://github.com/pik-piam/madrat).

5. R Core Team, *R: A Language and Environment for Statistical Computing* (Vienna, Austria, 2017; https://www.R-project.org/).

6. H. Lotze-Campen, C. Müller, A. Bondeau, S. Rost, A. Popp, W. Lucht, Global food demand, productivity growth, and the scarcity of land and water resources: a spatially explicit mathematical programming approach. *Agric. Econ.* **39**, 325–338 (2008).

7. J. P. Dietrich, B. L. Bodirsky, I. Weindl, F. Humpenöder, M. Stevanovic, U. Kreidenweis, X. Wang, K. Karstens, A. Mishra, D. Klein, G. Ambrósio, E. Araújo, A. Biewald, H. Lotze-Campen, A. Popp, *MAgPIE - An Open Source land-use modeling framework* (Potsdam Institute for Climate Impact Research, Potsdam, Germany, 2018; http://doi.org/10.5281/zenodo.1418752).

8. J. P. Dietrich, B. L. Bodirsky, F. Humpenöder, I. Weindl, M. Stevanović, K. Karstens, U. Kreidenweis, X. Wang, A. Mishra, D. Klein, G. Ambrósio, E. Araujo, A. W. Yalew, L. Baumstark, S. Wirth, A. Giannousakis, F. Beier, D. M.-C. Chen, H. Lotze-Campen, A. Popp, MAgPIE 4 - A modular open source framework for modeling global land-systems. *Geosci. Model Dev. Discuss.*, 1–26 (2018).

9. E. Kriegler, N. Bauer, A. Popp, F. Humpenöder, M. Leimbach, J. Strefler, L. Baumstark, B. L. Bodirsky, J. Hilaire, D. Klein, I. Mouratiadou, I. Weindl, C. Bertram, J.-P. Dietrich, G. Luderer, M. Pehl, R. Pietzcker, F. Piontek, H. Lotze-Campen, A. Biewald, M. Bonsch, A. Giannousakis, U. Kreidenweis, C. Müller, S. Rolinski, A. Schultes, J. Schwanitz, M. Stevanovic, K. Calvin, J. Emmerling, S. Fujimori, O. Edenhofer, Fossil-fueled development (SSP5): An energy and resource intensive scenario for the 21st century. *Glob. Environ. Change*. **42**, 297–315 (2017).

10. FAOSTAT, Database collection of the Food and Agriculture Organization of the United Nations (2015), (available at www.faostat.fao.org).

11. WHO, Global Health Observatory data repository (2018), (available at http://apps.who.int/gho/data/view.main.2487?lang=en).

12. NCD-RisC, Worldwide trends in body-mass index, underweight, overweight, and obesity from 1975 to 2016: a pooled analysis of 2416 population-based measurement studies in 128·9 million children, adolescents, and adults. *The Lancet*. **390**, 2627–2642 (2017).

13. NCD-RisC, A century of trends in adult human height. *eLife*. **5**, e13410 (2016).

14. FAO/WHO/UNU, *Human energy requirements* (2004; http://www.fao.org/tempref/docrep/fao/007/y5686e/y5686e00.pdf).

15. A. W. Froehle, Climate variables as predictors of basal metabolic rate: new equations. *Am. J. Hum. Biol. Off. J. Hum. Biol. Counc.* **20**, 510–529 (2008).

16. S. Kc, W. Lutz, The human core of the shared socioeconomic pathways: Population scenarios by age, sex and level of education for all countries to 2100. *Glob. Environ. Change*. **42**, 181–192 (2017).

17. IIASA, SSP Database (2015), (available at https://secure.iiasa.ac.at/web-apps/ene/SspDb/).

18. WORLDBANK, World Development Indicators (2013), (available at http://data.worldbank.org/data-catalog/world-development-indicators).

19. S. L. James, P. Gubbins, C. J. Murray, E. Gakidou, Developing a comprehensive time series of GDP per capita for 210 countries from 1950 to 2015. *Popul. Health Metr.* **10**, 12 (2012).

20. W. Lutz, A. Goujon, S. K.C., W. Sanderson, Reconstruction of populations by age, sex and level of educational attainment for 120 countries for 1970-2000. *Vienna Yearb. Popul. Res.* **5**, 193–235 (2007).

21. S. KC, W. Lutz, The human core of the shared socioeconomic pathways: Population scenarios by age, sex and level of education for all countries to 2100. *Glob. Environ. Change*, doi:10.1016/j.gloenvcha.2014.06.004.

22. E. Borghi, C. Casanovas, A. Onyango, “Stunting Policy Brief” (WHO, Geneva, Switzerland, 2014).

23. V. Smil, *Feeding the World: A Challenge for the 21st century* (MIT, 2000).

24. P. C. Hallal, L. B. Andersen, F. C. Bull, R. Guthold, W. Haskell, U. Ekelund, L. P. A. S. W. Group, Global physical activity levels: surveillance progress, pitfalls, and prospects. *The lancet*. **380**, 247–257 (2012).

25. P. J. M. Weijs, H. M. Kruizenga, A. E. van Dijk, B. S. van der Meij, J. A. E. Langius, D. L. Knol, R. J. M. S. van Schijndel, M. A. E. van B. van der Schueren, Validation of predictive equations for resting energy expenditure in adult outpatients and inpatients. *Clin. Nutr.* **27**, 150–157 (2008).

26. FAO, WHO, UNU, Energy and protein requirements. *Necesidades de energía y de proteínas : informe de una Reunión Consultiva Conjunta FAO/OMS/UNU de Expertos, [Roma, 5-17 de octubre de 1981]* (1985) (available at http://www.who.int/iris/handle/10665/39527).

27. W. N. Schofield, Predicting basal metabolic rate, new standards and review of previous work. *Hum. Nutr. Clin. Nutr.* **39 Suppl 1**, 5–41 (1985).

28. WHO, *WHO child growth standards: length/height-for-age, weight-for-age, weight-for-length, weight-for-height and body mass index-for-age ; methods and development* (WHO Press, Geneva, 2006).

29. M. de Onis, Development of a WHO growth reference for school-aged children and adolescents. *Bull. World Health Organ.* **85**, 660–667 (2007).

30. J. Gustavsson, C. Cedersberg, U. Sonesson, “Global Food Losses and Food Waste” (FAO, Düsseldorf, 2011).

31. C. Weyer, R. L. Walford, I. T. Harper, M. Milner, T. MacCallum, P. A. Tataranni, E. Ravussin, Energy metabolism after 2 y of energy restriction: the biosphere 2 experiment. *Am. J. Clin. Nutr.* **72**, 946–953 (2000).

32. M. Sun, B. A. Gower, T. R. Nagy, C. A. Trowbridge, C. Dezenberg, M. I. Goran, Total, resting, and activity-related energy expenditures are similar in Caucasian and African-American children. *Am. J. Physiol.* **274**, E232-237 (1998).

33. M. W. Vander Weg, R. C. Klesges, K. D. Ward, Differences in resting energy expenditure between black and white smokers: implications for postcessation weight gain. *Eur. J. Clin. Nutr.* **54**, 895–899 (2000).

34. J. L. Waid, M. Ali, S. H. Thilsted, S. Gabrysch, Dietary change in Bangladesh from 1985 to 2010. *Glob. Food Secur.* **17**, 221–232 (2018).

35. L. C. Del Gobbo, S. Khatibzadeh, F. Imamura, R. Micha, P. Shi, M. Smith, S. S. Myers, D. Mozaffarian, Assessing global dietary habits: a comparison of national estimates from the FAO and the Global Dietary Database. *Am. J. Clin. Nutr.* **101**, 1038–1046 (2015).

36. Insee, www.insee.fr (2018), (available at https://www.insee.fr/fr/accueil).

37. CIA, “The World Factbook 2018” (Central Intelligence Agency, Washington, DC, 2018).

38. Worldometer, Population by country (2018), (available at https://www.worldometers.info/world-population/population-by-country/).

39. Statistics for Development of the Pacific Community, Prism (2018), (available at https://prism.spc.int/).

40. BEA, GDP for the U.S. Territories (2018), (available at https://www.bea.gov/data/gdp/gdp-us-territories).

41. N. Alexandratos, J. Bruinsma, “World agriculture towards 2030/2050: the 2012 revision” (ESA Working paper Rome, FAO, 2012), (available at http://environmentportal.in/files/file/World%20agriculture%20towards%202030.pdf).

42. D. L. Bijl, P. W. Bogaart, S. C. Dekker, E. Stehfest, B. J. M. de Vries, D. P. van Vuuren, A physically-based model of long-term food demand. *Glob. Environ. Change*. **45**, 47–62 (2017).

43. C. Le Mouël, A. Forslund, How can we feed the world in 2050? A review of the responses from global scenario studies. *Eur. Rev. Agric. Econ.* **44**, 541–591 (2017).

44. V. Huber, I. Neher, B. L. Bodirsky, K. Höfner, H. J. Schellnhuber, Will the world run out of land? A Kaya-type decomposition to study past trends of cropland expansion. *Environ. Res. Lett.* **9**, 024011 (2014).

45. M. Springmann, M. Clark, D. Mason-D’Croz, K. Wiebe, B. L. Bodirsky, L. Lassaletta, W. de Vries, S. J. Vermeulen, M. Herrero, K. M. Carlson, M. Jonell, M. Troell, F. DeClerck, L. J. Gordon, R. Zurayk, P. Scarborough, M. Rayner, B. Loken, J. Fanzo, H. C. J. Godfray, D. Tilman, J. Rockström, W. Willett, Options for keeping the food system within environmental limits. *Nature* (2018), doi:10.1038/s41586-018-0594-0.

46. J. G. Conijn, P. S. Bindraban, J. J. Schröder, R. E. E. Jongschaap, Can our global food system meet food demand within planetary boundaries? *Agric. Ecosyst. Environ.* **251**, 244–256 (2018).

47. D. Gerten, V. Heck, J. Jägermeyr, B. L. Bodirsky, I. Fetzer, M. Jalava, M. Kummu, W. Lucht, J. Rockström, S. Schaphoff, H. J. Schellnhuber, Feeding ten billion people is possible within four terrestrial planetary boundaries. *Nat. Sustain.*, 1–9 (2020).

48. B. L. Bodirsky, thesis, TU Berlin, Berlin, Germany (2014).

49. R. C. Henry, K. Engström, S. Olin, P. Alexander, A. Arneth, M. D. A. Rounsevell, Food supply and bioenergy production within the global cropland planetary boundary. *PLOS ONE*. **13**, e0194695 (2018).

50. H. Kahiluoto, M. Kuisma, A. Kuokkanen, M. Mikkilä, L. Linnanen, Taking planetary nutrient boundaries seriously: Can we feed the people? *Glob. Food Secur.* **3**, 16–21 (2014).

51. M. Stevanović, A. Popp, B. L. Bodirsky, F. Humpenöder, C. Müller, I. Weindl, J. P. Dietrich, H. Lotze-Campen, U. Kreidenweis, S. Rolinski, A. Biewald, X. Wang, Mitigation Strategies for Greenhouse Gas Emissions from Agriculture and Land-Use Change: Consequences for Food Prices. *Environ. Sci. Technol.* **51**, 365–374 (2017).

52. E. Stehfest, A. F. Bouwman, D. P. Vuuren, M. G. J. Elzen, B. Eickhout, P. Kabat, Climate benefits of changing diet. *Clim. Change*. **95**, 83–102 (2009).

53. P. Smith, H. Haberl, A. Popp, K. Erb, C. Lauk, R. Harper, F. N. Tubiello, A. de Siqueira Pinto, M. Jafari, S. Sohi, O. Masera, H. Böttcher, G. Berndes, M. Bustamante, H. Ahammad, H. Clark, H. Dong, E. A. Elsiddig, C. Mbow, N. H. Ravindranath, C. W. Rice, C. Robledo Abad, A. Romanovskaya, F. Sperling, M. Herrero, J. I. House, S. Rose, How much land-based greenhouse gas mitigation can be achieved without compromising food security and environmental goals? *Glob. Change Biol.* **19**, 2285–2302 (2013).
